# Disruption of ACTG1 potentiates anti-TIM-3 immunotherapy by reprogramming the immunosuppressive microenvironment via the CSF3/CSF3R axis in pancreatic cancer

**DOI:** 10.3389/fimmu.2026.1820470

**Published:** 2026-10-14

**Authors:** Le Zhu, Yu Guo, Xinxin Ling, Yuqi Tao, Shun Wang, Sanfeng Han, Meiping Ni, Huarong Mao, Jiajun Wu, Chengjie Zhang, Ziping Zhang, Qimeng Chang, Zhiqiu Hu, Junmin Chen, Bingsheng Sun, Xiaodi Yang, Qiongzhu Dong

**Affiliations:** 1 Key Laboratory of Whole-Period Monitoring and Precise Intervention of Digestive Cancer, Shanghai Municipal Health Commission (SMHC), Department of Hepatobiliary and Pancreatic Surgery, Minhang Hospital, Fudan University, Shanghai, China; 2 Department of General Surgery, Huashan Hospital and Cancer Metastasis Institute, Fudan University, Shanghai, China; 3 Guangzhou Institute of Cancer Research, The Affiliated Cancer Hospital, Guangzhou Medical University, Guangzhou, Guangdong, China; 4 College of Acupuncture-moxibustion, Anhui University of Chinese Medicine, Hefei, Anhui, China; 5 Traditional Chinese Medicine Department, Minhang Hospital, Fudan University, Shanghai, China; 6 Department of Pulmonary Oncology, Tianjin Medical University Cancer Institute and Hospital, National Clinical Research Center for Cancer, Tianjin’s Clinical Research Center for Cancer, Key Laboratory of Cancer Prevention and Therapy, Tianjin, China

**Keywords:** ACTG1, pancreatic ductal adenocarcinoma, prognosis, TIM-3, tumor microenvironment

## Abstract

**Background:**

The limited immune infiltration observed in pancreatic ductal adenocarcinoma (PDAC) is a major factor contributing to immunotherapy resistance and the consistently poor prognosis. Hence, identifying novel therapeutic targets and developing effective combination strategies to overcome immunotherapy resistance represent urgent and unmet clinical needs.

**Methods:**

The gene expression datasets and corresponding clinical information were collected from TCGA, GEO and ICGC. The immune cell infiltration (ICI) contents were evaluated by “CIBERSORT”. PDAC subtypes based on ICI pattern were obtained from consensus clustering. Their prognostic value was validated by the Kaplan-Meier survival curves. Then, we employed an integrated multi-dataset approach to systematically identify pivotal regulators of PDAC progression. The functional role of ACTG1 was specifically validated through *in vivo* and *in vitro* models. The tumor immune microenvironment was characterized by flow cytometry and immunohistochemistry (IHC). Potential molecular mechanisms were further investigated using RNA sequencing and tumor cell-macrophage co-culture experiments.

**Results:**

Three distinct immune infiltration subtypes with different prognoses and immunological features were identified in the 608-sample GEO/ICGC training cohort and the 177-sample TCGA-PAAD validation cohort. The ICI pattern with a better prognosis was characterized by high level of plasma cells, activated NK cells, activated DC cells, M1 macrophages and CD8^+^ T cells, low level of M0 macrophages, M2 macrophages. ACTG1 drives immunosuppression and predicts poor prognosis in PDAC. Mechanistically, ACTG1 promotes tumor cell-derived CSF3 secretion, which upregulates TIM-3 expression on tumor-associated macrophages (TAMs) via the CSF3/CSF3R signaling pathway. This cascade reinforces M2-like TAMs polarization and induces CD8^+^ T cell exhaustion, thereby fostering an immunosuppressive niche. Strikingly, ACTG1 knockdown combined with TIM-3 blockade exerts an enhanced effect in reversing immunosuppression, restoring anti-tumor immunity, and markedly prolonging survival.

**Conclusions:**

ACTG1 promotes PDAC progression by orchestrating an immunosuppressive tumor microenvironment through the CSF3/CSF3R/TIM-3 axis. Thus, combining genetic suppression of ACTG1 with TIM-3 blockade represents a potent therapeutic approach to overcome immune evasion and curb tumor progression.

## 1 Introduction

Pancreatic ductal adenocarcinoma (PDAC) accounts for approximately 90% of all pancreatic cancers and ranks among the most lethal malignancies, with a five-year survival rate below 12%o (1). Most patients are diagnosed at an advanced stage, with local invasion or distant metastasis, rendering current therapeutic options largely ineffective and rarely curative (2). Although combination regimens such as FOLFIRINOX, gemcitabine/nab-paclitaxel, and nanoliposomal irinotecan/fluorouracil can extend survival to some extent, their efficacy remains limited (3). These challenges underscore the urgent need to elucidate the mechanisms driving PDAC progression and to identify novel therapeutic targets for this highly aggressive cancer.

PDAC is characterized by a highly immunosuppressive tumor microenvironment (TME), marked by diminished effector T cell infiltration and the accumulation of immunosuppressive cells such as tumor-associated macrophages (TAMs) (4, 5). Within this context, immunosuppressive myeloid cells are recruited to the tumor site via chemokines and other signaling molecules, where they adopt a phenotype that promotes immune evasion and disease progression (6–8). Among the key regulators of this process is the immune checkpoint molecule TIM-3, which is expressed on a variety of immune cells including monocytes, macrophages, and T lymphocytes. By coordinating multiple immune cell populations and promoting the secretion of immunosuppressive chemokines and cytokines, TIM-3 reinforces the immunosuppressive TME and drives tumor progression. Importantly, targeting TIM-3 has been shown to shift the TME from an immunosuppressive to an inflammatory state (9, 10). Cytokines also play a critical role in shaping the TME. For example, granulocyte colony-stimulating factor (CSF3) and its receptor (CSF3R) are elevated in tumor tissues compared to normal tissues. CSF3 can impair IFN-γ production by T cells, and anti-CSF3 treatment has been demonstrated to modulate T cell infiltration, activation, and IFN-γ responses (11). Together, these findings highlight the potential of targeting chemokine-receptor interactions to disrupt immunosuppressive cell recruitment and reactivate antitumor immunity, offering a promising approach to reprogram the TME in PDAC.

ACTG1 (γ-actin 1) is a core component of the cytoskeleton and regulates multiple biological processes including mechanical stress, cell motility, and division. Recent studies have shown that aberrant expression of ACTG1 enhances tumor cell migration, invasion, and progression (4–6). It has been reported that ACTG1 binds to myosin 9 to form a complex that promotes nuclear translocation of oncogenes, thereby exacerbating malignant phenotypes (5). Moreover, exosome-derived PGAM1 interacts with ACTG1 to stimulate pseudopodia formation and neovascular sprouting in human umbilical vein endothelial cells (HUVECs), ultimately accelerating tumor progression. High ACTG1 expression correlates with adverse prognosis (6, 7) and is involved in tumor microenvironment modulation (7). Nevertheless, its precise function in facilitating tumor-immune communication is still poorly understood.

In this study, we demonstrated that ACTG1 is simultaneously associated with both prognosis and immune regulation in pancreatic cancer. ACTG1 promotes M2 polarization of macrophages through the CSF3/CSF3R/TIM-3 signaling axis, thereby inducing an immunosuppressive tumor microenvironment. Importantly, combined targeting of ACTG1 and TIM-3 significantly improved survival in PDAC models, providing a novel theoretical foundation for expanding immunotherapy strategies.

## 2 Methods

### 2.1 Sample collection and bioinformatic analysis

A total of 785 pancreatic ductal adenocarcinoma (PDAC) bulk transcriptomic samples were included in this study, which were divided into a training cohort (n = 608) and an independent validation cohort (n = 177). The training cohort comprised 205 samples from the Gene Expression Omnibus (GEO) database (accession numbers: GSE71729 and GSE85916; microarray data) and 403 samples from the International Cancer Genome Consortium (ICGC) database (projects: PACA-AU and PACA-CA; bulk RNA-seq raw gene read counts). The TCGA-PAAD cohort (n = 177; bulk RNA-seq raw gene read counts) from The Cancer Genome Atlas was used as the independent validation cohort.

To control for cohort heterogeneity, we first performed rigorous quality control on all samples: samples with >30% missing gene expression values were excluded, and patients with incomplete survival status or survival time information were also removed from subsequent analyses. Raw read counts obtained from ICGC were uniformly converted to Fragments Per Kilobase of transcript per Million mapped reads (FPKM) to normalize for differences in gene length and sequencing depth across samples. This conversion was performed using the fpkm() function from the DESeq2 R package (v1.42.0) based on GENCODE v38 gene annotation. Subsequently, all FPKM values were log2-transformed using the formula log_2_(FPKM + 1) to stabilize variance, approximate a normal distribution suitable for downstream statistical analyses, and avoid mathematical errors caused by log-transformation of zero expression values.

Following normalization, we applied the ComBat algorithm to correct for technical batch effects across the 608 training cohort samples. We adjusted for known technical covariates including sequencing platform, library preparation protocol, and sequencing center, while preserving biological variation associated with immune subtypes and clinical outcomes. The same normalization pipeline (FPKM conversion followed by log2 transformation) was applied to the 177-sample TCGA validation cohort to ensure methodological consistency between the two cohorts. No additional batch correction was performed on the validation cohort to maintain its independence for subtype stability validation.

Subsequently, we used the ESTIMATE, CIBERSORT, and EPIC algorithms to calculate immune infiltration scores and characterize the immune landscape of PDAC tumors in both the training and validation cohorts. Unsupervised consensus clustering was performed using the ConsensusClusterPlus R package to identify distinct immune subtypes based on the immune infiltration profiles.

### 2.2 Cell cultures

The HEK 293T cell line, along with mouse pancreatic cancer cell line (Panc02) and murine monocyte/macrophage lines RAW 264.7 and J774A.1, were obtained from the Institute of Biochemistry and Cell Biology at the Chinese Academy of Sciences (Shanghai, China). The 49016 (LSL-Kras^G12D/+^, p53^R172H/+^, p48^Cretg/+^) cell line was donated by the team of Bo Kong at Heidelberg University Hospital, Heidelberg, Germany. All cell lines were cultured in Dulbecco’s Modified Eagle Medium (DMEM; Gibco) supplemented with 10% fetal bovine serum (FBS; Gibco), and maintained at 37.0 °C in a humidified incubator with 5% CO_2_.

### 2.3 RNA sequencing

RNA sequencing was conducted by Wuhan IGENEBOOK Biotechnology Co., Ltd. Total RNA was isolated with the RNAprep Pure Kit (DP432; TIANGEN) according to the manufacturer’s protocol. The integrity of all RNA samples was assessed using a Qsep400 instrument. Sequencing libraries were constructed from 1 μg of total RNA per sample using the VAHTS mRNA-seq V8 Library Prep Kit for Illumina (12). The workflow involved polyA-selected RNA extraction, fragmentation, reverse transcription with random hexamers, and finally, 150 bp paired-end sequencing on an Illumina Novaseq 6000 platform.

### 2.4 Bioinformatics analysis of RNA-seq

Data processing began with the removal of adapter sequences and low-quality reads using cutadapt (v1.11). The resulting clean reads were mapped to the *Mus musculus* reference genome via Hisat2 (v2.1.0) with a two-mismatch allowance. Following annotation against the NR protein database, transcript abundance was estimated by FeatureCounts (v1.6.0) and normalized as FPKM. The edgeR package was applied to identify differentially expressed genes under the criteria of FDR < 0.05 and |log_2_FoldChange| > 1. For functional characterization, GO and KEGG enrichment analyses were executed in R using ClusterProfiler, with a hypergeometric distribution test and a q-value cutoff of 0.05 to define significant enrichment.

### 2.5 Animal studies and treatment models

Male C57BL/6 mice, aged 6 weeks and weighing 20–25 g, were supplied by Shanghai Slac Laboratory Animal Co., Ltd. For all experiments involving mice were complied with NIH guidelines and were approved by the Institutional Animal Care and Use Committee of Fudan university (approval number: 202103017S).

The animals were allowed free access to standard rodent diet and water throughout the study. Before surgical interventions, anesthesia was induced by intraperitoneal administration of sodium pentobarbital (40 mg/kg; Sigma-Aldrich, St. Louis, MO, USA). All experimental procedures were performed in a specific pathogen-free (SPF) environment.

To establish orthotopic pancreatic tumors, we injected 2 × 10^6^ Panc02 or 49016 cells (including Scr, Sh-ACTG1, EV, and OE-ACTG1) in 100 μL PBS into the pancreas of C57BL/6 mice (5 mice/group). All tumors were subsequently harvested, and their weights recorded. Additionally, mouse anti-TIM-3 antibody (10 mg/kg, Bio X Cell, Cat#CD366, RRID: AB_ 2687798) or IgG control (Bio X Cell, Cat#BE0089, RRID: AB_1107769) was administered intraperitoneally (i.p.) twice weekly.

### 2.6 Plasmid and cell transfections

Stable ACTG1-overexpressing cells were generated by cloning the mouse ACTG1 cDNA (NP_033739.1) into a pCDH-CMV-MCS-EF1-puro lentiviral vector. The recombinant plasmid was then packaged into lentiviral particles by co-transfecting it with the psPAX2 and pMD2.G packaging plasmids into HEK293T cells using Lipofectamine 3000. Similarly, for stable knockdown, shRNAs targeting mouse ACTG1 (Target sequence 5’-3’; ACTG1-sh1: GACCACTTTCAACTCCATCAT; ACTG1-sh2: CACGATGAAGATTAAGATCAT) were cloned into a pLKO.1-puro vector and packaged into retroviruses by co-transfection of the construct with GAG-POL and VSVG plasmids into HEK293T cells.

### 2.7 Flow cytometry

Single-cell suspensions were prepared from orthotopic mouse tumors using a semi-automated dissociation protocol with the gentleMACS Dissociator and a Tumor Dissociation Kit (Miltenyi Biotec, Cat#130-096-730). The harvested cells were then subjected to red blood cell lysis using a commercial buffer (Tiangen, Cat#RT122). The resulting cell suspensions were blocked with a mouse FcR blocking reagent (Miltenyi Biotec, Cat#130-092-575, USA) and subsequently stained with antibodies targeting mouse CD45 (BD Biosciences, Cat#564279, RRID: AB_2651134), CD3 (BioLegend, Cat#100219, RRID: AB_1732068), CD4 (BioLegend, Cat#100552, RRID: AB_2563053), CD8 (BioLegend, Cat#100752, RRID: AB_2563057), Granzyme B (BioLegend, Cat#515409, RRID: AB_3106190), TIM-3 (BioLegend, Cat#119703, RRID: AB_345378), CD11b (BioLegend, Cat#101205, RRID: AB_312788), Gr-1 (BioLegend, Cat#108428, RRID: AB_893558), F480 (BioLegend, Cat#123115, RRID: AB_893493), CD206 (BioLegend, Cat#141717, RRID: AB_2562232), TGF-β (BioLegend, Cat#141403, RRID: AB_10730610), IL-10 (BioLegend, Cat#505009, RRID: AB_315363), as required by the experiment. For Granzyme B staining, cells were restimulated with 1 μL/mL Cell Activation Cocktail (with Brefeldin) in 5% CO_2_ for 5 hours at 37°C before intracellular staining. Fluorescence detection was carried out using BUV395 anti-mouse CD45, PE/Cy7 anti-mouse CD3, BV785 anti-mouse CD4, BV510 anti-mouse CD8, PE anti-mouse TIM-3, BV421 anti-mouse Granzyme B, FITC anti-mouse CD11b, PerCP/Cy5.5 anti-mouse Gr-1, APC anti-mouse F480, BV421 anti-mouse CD206, PE anti-mouse TGF-β and APC anti-mouse IL-10. All samples were acquired on a BD FACSVerse™ (BD Biosciences, USA) flow cytometer and the resulting data were analyzed with FlowJo software (BD Biosciences, USA).

### 2.8 IHC of pancreatic cancer tissues

Following deparaffinization and rehydration, tissue sections were subjected to antigen retrieval to unmask the target epitopes. The sections were then incubated with specific primary antibodies at 4°C overnight to facilitate binding. The next day, a secondary antibody (Dako Denmark A/S, Glostrup, Denmark) was applied and the sections were incubated for 30 minutes at 37°C. Antibody binding was visualized using a 3,3’-diaminobenzidine (DAB) substrate, which yields a brown precipitate at the antigen-positive sites. Finally, hematoxylin counterstaining was performed to provide morphological context for observation by light microscopy. The primary antibodies employed in this study were: ACTG1 (Proteintech, Cat#11227-1-AP, RRID: AB_2223507), IFN-γ (Proteintech, Cat#29788-1-AP, RRID: AB_3086161), CD206 (CST, Cat#24595T, RRID: AB_2892682), TIM-3 (CST, Cat# 83882T, RRID: AB_2800033), and CSF3 (ABclonal, Cat#A6178, RRID: AB_2766794).

Following staining, whole-slide images were acquired using a digital pathology scanner under identical exposure, brightness, and contrast settings to ensure consistency across all samples. Quantitative analysis of DAB staining intensity was performed using ImageJ software (NIH, Bethesda, MD, USA). Briefly, uniform background correction was applied to all images prior to analysis. For each tissue section, 5 representative tumor regions were randomly selected as regions of interest (ROIs), avoiding areas of necrosis, hemorrhage, or normal tissue. The integrated optical density (IOD) and area of each ROI were measured, and the mean optical density (IOD/Area) was calculated as an indicator of protein expression level. To facilitate data visualization and comparison, the mean optical density values were normalized to a 0–5 scale, where 0 represented the lowest expression level and 5 represented the highest expression level across all samples. All quantitative analyses were performed independently by two pathologists in a blinded manner, and the average score was used for statistical analysis.

### 2.9 Enzyme-linked immunosorbent assay

Pancreatic cancer cell lines were seeded in six-well plates at a density of 5 × 10^5^ cells per well. After 48 hours of culture, the supernatants were collected and the concentration of CSF3 was quantified using a Mouse CSF3 ELISA Kit (MULTI SCIENCES, Cat# EK269) according to the manufacturer’s instructions.

### 2.10 Immunofluorescence

For immunofluorescence staining, cells were fixed in 4% paraformaldehyde (20 min, RT), permeabilized with 0.5% Triton X-100 (15 min), and blocked with 1% BSA for 1 hour. Primary antibody incubation was performed targeting TIM-3 (Proteintech, Cat# 60355-1-Ig, RRID: AB_2881464), followed by detection with an Alexa Fluor 488-conjugated anti-mouse secondary antibody (Life Technologies). Nuclei were visualized with DAPI. Finally, the specimens were mounted and visualized using an Olympus confocal microscope (Olympus, Tokyo, Japan).

### 2.11 Co-culture assays

We employed an indirect co-culture model to study macrophage-PDAC cell interactions using Raw264.7 and J774A.1 macrophages alongside 49016 and 49016-OE-ACTG1 PDAC cells. Macrophages were plated in 6-well plates (5 × 10^5^ cells/well), and PDAC cells were added in transwell inserts (0.4 μm pore; Corning) at a 1:1 ratio. After 48 hours of co-culture, macrophages were analyzed by flow cytometry for polarization markers (IL-10, TGF-β). TIM-3 expression and localization were additionally evaluated by Western blotting and immunofluorescence. The co-culture systems were additionally supplemented with the following reagents: a neutralizing anti-CSF3 antibody (R&D Systems, Cat# MAB414, RRID: AB_2085954), a blocking anti-CSF3R antibody (R&D Systems, Cat# MAB6039, RRID: AB_10890232), isotype anti-IgG antibody (R&D Systems, Cat# MAB005, RRID: AB_357348) and recombinant CSF3 protein (R&D Systems, Cat# 414-CS).

### 2.12 Western blot

Total protein was extracted by lysing cells in RIPA buffer containing a protease inhibitor cocktail. The lysates were then resolved by SDS-PAGE and electrotransferred onto polyvinylidene fluoride (PVDF) membranes. After blocking with 5% non-fat milk in TBST, the membranes were incubated with primary antibodies at 4°C overnight, followed by a corresponding horseradish peroxidase (HRP)-conjugated secondary antibody for 1 hour at room temperature. The following primary antibodies were used: ACTG1 (Proteintech, Cat#11227-1-AP, RRID: AB_2223507), TIM-3 (CST, Cat# 83882T, RRID: AB_2800033) and β-actin (Proteintech, Cat#60008-1-Ig, RRID: AB_2289225). Protein bands were visualized using the ImageQuant™ LAS 4000 imaging system (GE Healthcare Life Sciences).

### 2.13 Data availability

A training cohort of 608 and validation cohort of 177 PDAC samples was utilized for bulk RNA-seq analysis. The dataset included 205 samples sourced from the GEO database (GSE71729, GSE85916), 403 samples from the ICGC database (ICGC-PAAD-AU, ICGC-PAAD-CA) and 177 samples from the TCGA database. The Kaplan-Meier plotter website provides data for 278 PDAC samples (https://doi.org/10.1111/cts.13563). Additional data relevant to this study can be obtained from the Lead Contact (qzhdong@fudan.edu.cn) upon reasonable request.

### 2.14 Statistical analysis

All data are expressed as the mean ± SEM. GraphPad Prism^®^ 8.0 was used to perform all statistical analyses. Survival curves were generated by the Kaplan-Meier method and compared using the log-rank test. Differences between groups were assessed by Student’s t-test, one-way ANOVA, or two-way ANOVA, as appropriate. A 95% confidence interval was applied to determine statistical significance, with the following thresholds: ns, not significant (*p* > 0.05); *, *p* < 0.05; **, *p* < 0.01; ***, *p* < 0.001.

Further materials and methods are provided in the **Supplementary Materials** and methods.

## 3 Results

### 3.1 The landscape of immune cell infiltration in PDAC

To characterize the immune landscape of the PDAC tumor microenvironment, we analyzed 608 PDAC samples in the training cohort (205 from GEO database, 403 from ICGC database) after ComBat batch correction (**Figure 1A**; **Supplementary Figure 1A**). Immune infiltration was assessed using the ESTIMATE, CIBERSORT and EPIC algorithms (**Supplementary Tables S1-S3**). Using the R package “ConsensusClusterPlus”, we classified the PDAC samplesinto distinct subgroups based on ICI profiles. The optimal number of clusters was determined to be K = 3, which exhibited the highest stability according to cumulative distribution function analysis (**Supplementary Figure 1B**; **Supplementary Table S4**). A comprehensive heatmap was generated to visualize the ICI patterns alongside clinical characteristics (**Figure 1B**). Kaplan-Meier survival analysis revealed significant differences among the three ICI clusters: ICI-C1 cluster was associated with the longest median survival, whereas ICI-C2 cluster exhibited the worst prognosis (**Figure 1C**, *p* = 0.0036). To further explore the relationship between immune scores and infiltrating immune cells, we generated box plots illustrating the tumor microenvironment landscape across the three ICI clusters in PDAC (**Figure 1D**). Patients from ICI-C1 cluster exhibited high infiltration of both immune cells and immunescore. In details, ICI-C1 cluster indicated high level of activated dendritic cells, monocytes, activated NK cells and CD8^+^ T cells. A multitude of experiments showed that NK cells and CD8^+^ T cells could reverse the immunosuppressive phenotype and played an enormous role in antitumor immune response (13, 14). Cluster ICI-C2 was associated with a high level of naïve B cells, Eosinophils, M0 Macrophages, activated mast cells, resting memory CD4^+^ T cells and lowest level of immune score. Abundant evidences highlighted that high infiltration of B cells, M0 macrophages, and M2 macrophages maybe display suppressive immunity and more aggressive phenotypes, which are detrimental to immunotherapy (14). To some extent, ICI-C3 cluster was partially similar to ICI-C1 cluster. Among them, these subpopulations such as activated DCs, activated NK cells and CD8^+^ T cells displayed high level. Importantly, ICI-C3 was also found to be characterized by high level of M2 macrophages, plasma cells and stroma score, which could interpret this bad prognosis in ICI-C3 cluster. Furthermore, correlation analysis among immune cells in 608 PDAC patients revealed that immune score generally showed positive correlations with immune-activated cell populations, whereas negative correlations were observed with immunosuppressive cell populations (**Supplementary Figure 1C**). In addition, we validated the ICI clustering in the independent TCGA-PAAD cohort andobserved immune infiltration patterns consistent with the training cohort (**Supplementary Figure 2A**). The ICI-C1 cluster showed high immune cell infiltration and elevated immune scores, withenrichment of resting mast cells, activated NK cells, and CD8^+^ T cells. In contrast,ICI-C2 was characterized by high naïve B cells and M2 macrophages with the lowest immune scores, while ICI-C3 was enriched in M0 macrophages. Patients in ICI-C1 also had significantly superior overall survival compared with the other two subtypes (**Supplementary Figures 2B, C**). This combined immune infiltration subtyping and prognostic analysis serves as a reliable tool for clinical risk stratification and guides the exploration of new immunotherapeutic targets.

**Figure 1:**
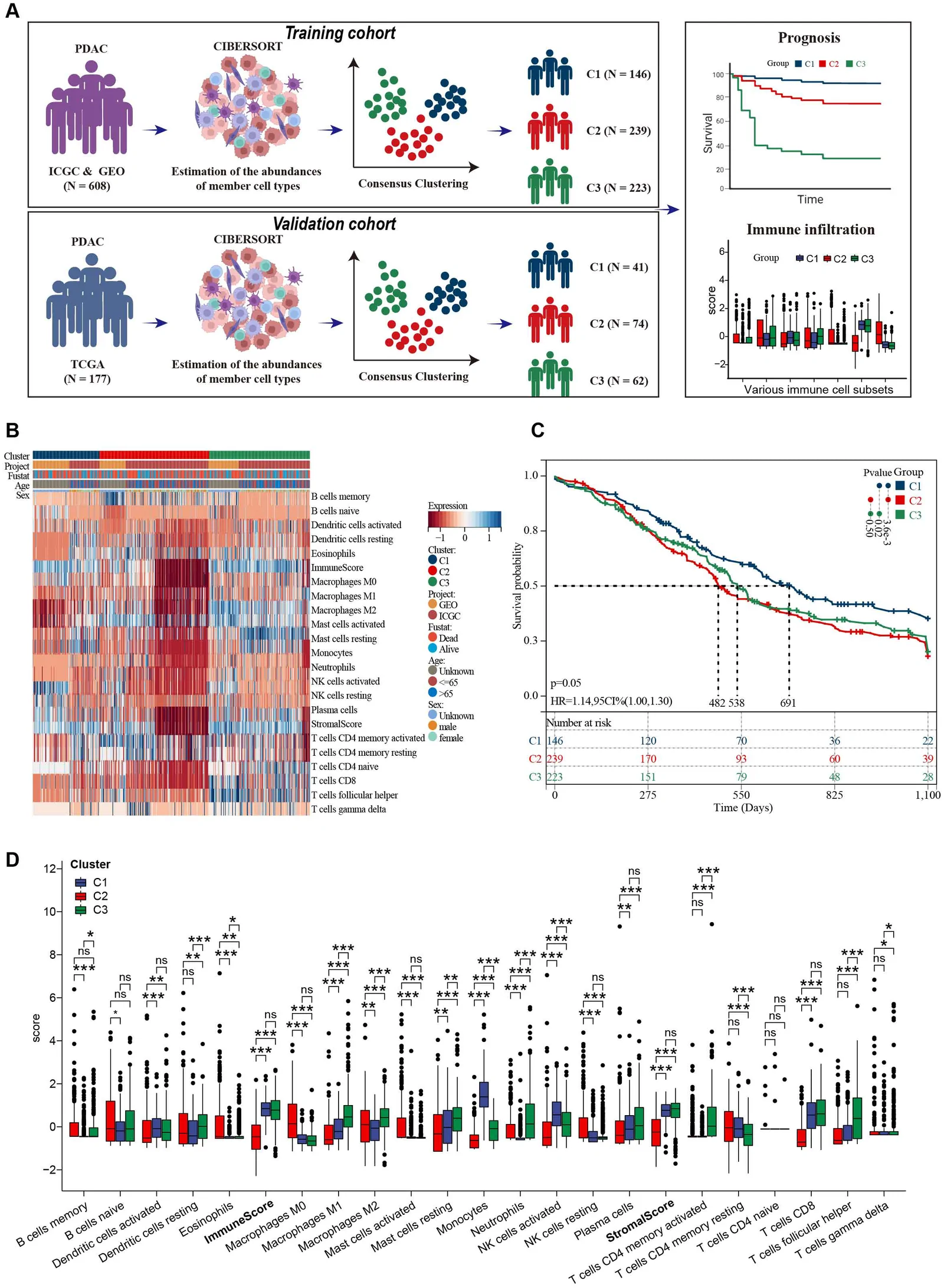
The immune infiltration landscape in PDAC patients as characterized by CIBERSORT algorithm. **(A)** Flowchart showing immune subtyping of 608 training and 177 validation PDAC patients based on immune infiltration patterns. Tumor microenvironment composition was estimated by CIBERSORT using GEO, ICGC, and TCGA data, followed by clustering to identify immune subtypes and assess their prognostic value. **(B)** Heatmap of tumor-infiltrating immune cell proportions and clinical features in training PDAC cohort. **(C)** Survival curves were generated using the Kaplan-Meier method, and the log-rank test was applied to assess overall survival differences among the three different ICI clusters. For pairwise comparisons between groups, p-values were adjusted using the Bonferroni method to control the family-wise error rate (FWER). **(D)** The fraction of tumor-infiltrating immune cells, immune score and stroma score across the three ICI clusters in the PDAC training cohort. Data represent mean ± SEM. To evaluate statistical significance, log-rank test with Bonferroni adjustment was performed to compare survival curves **(C)**, One- or two-way ANOVA was utilized to compare two variables **(D)**. *p < 0.05; **p < 0.01, ***p < 0.001.

### 3.2 Integrating multi-parameter immune profiling for prognostic stratification in PDAC

We further picked four immune checkpoints blockade (ICB)-related genes (PD-L1, CTLA4, LAG3 and TIM3) and two immune-activated genes (CD40LG and GZMB) (15–17). Then, we compared the expression level of these genes in the three ICI clusters. ICI-C1 cluster presented the lowest expression level of ICB-related genes, and our results showed that ICI-C2 cluster possessed the highest expression level of four ICB-related genes, and the lowest expression level of two immune-activated genes (**Figures 2A–F**).

**Figure 2:**
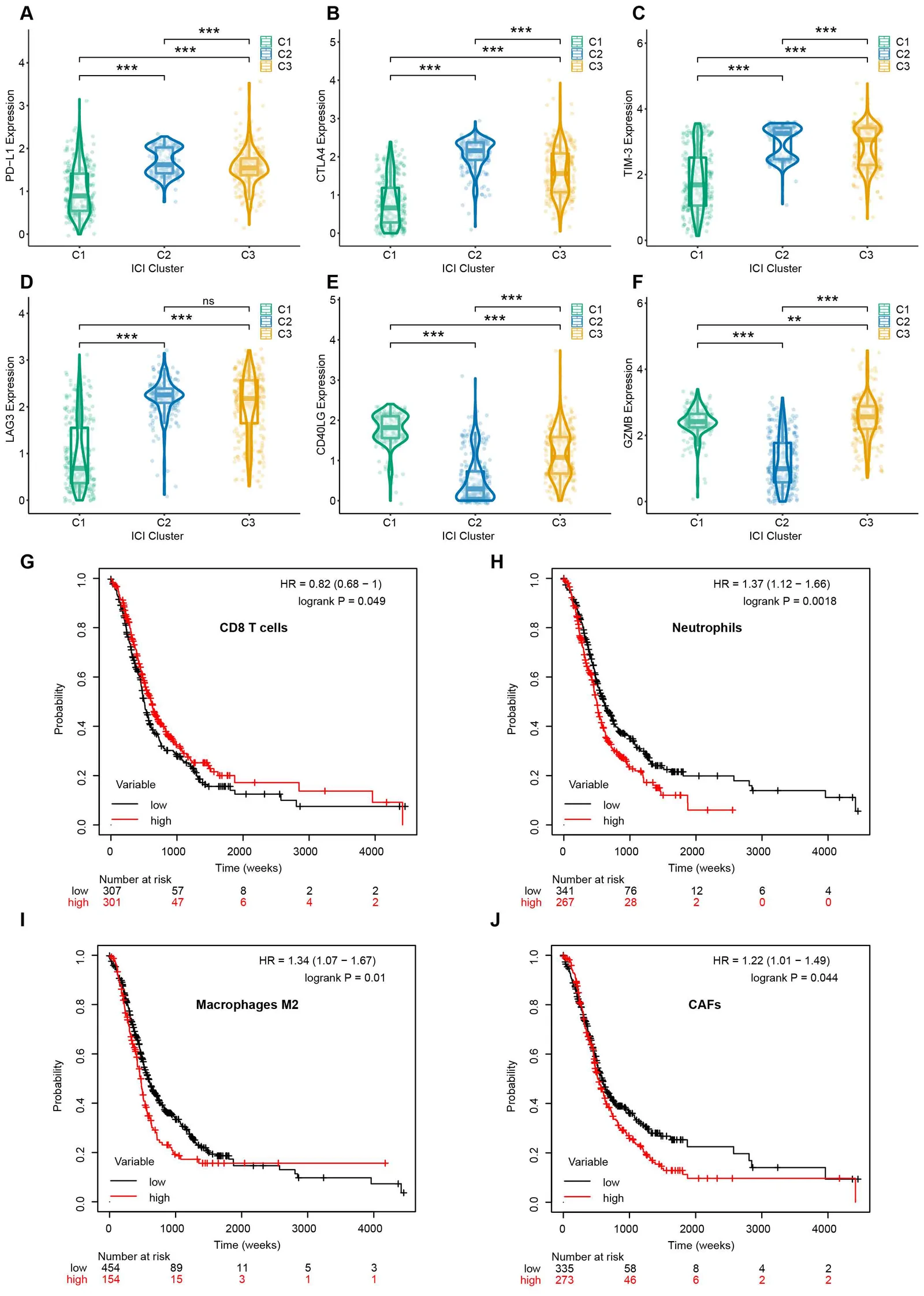
Analysis of exhaustion and cytotoxicity markers in three ICI clusters, and the prognostic significance of immune and stroma cell infiltration in PDAC. **(A–F)** The difference of PD-L1 **(A)**, CTLA-4 **(B)**, TIM-3 **(C)**, LAG-3 **(D)**, CD40LG **(E)** and GZMB **(F)** expression among distinct ICI clusters. **(G–J)** Prognostic significance of high versus low expression levels of specific immune or stroma cells in 608 PDAC patients. Patients were stratified based on the infiltration scores of CD8^+^ T cells **(G)**, Neutrophils **(H)**, M2 Macrophages **(I)** and CAFs **(J)**, which were calculated from tumor immune infiltration analysis using the EPIC and CIBERSORT algorithms. Data represent mean ± SEM. To evaluate statistical significance, One- or two-way ANOVA was utilized to compare two variables **(A–F)**, log-rank test with Bonferroni adjustment was performed to compare survival curves **(G–J)**. **p < 0.01, ***p < 0.001.

To further estimate the prognostic value of ICI, stratification survival analysis was performed based on previously classified ICI subpopulations. Infiltration of CD8^+^ T cells was discovered to be significantly correlated with a longer survival (**Figure 2G**), whereas a high percentage of Neutrophils and M2 macrophages exhibited a poor survival prognosis (**Figures 2H, I**). Additionally, the EPIC algorithm was performed to evaluate the percentage of immune subpopulation, and we found that a low percentage of cancer-associated fibroblasts (CAFs) subpopulation was associated with a good survival prognosis (**Figure 2J**).

Collectively, these results indicate that an immunosuppressive tumor microenvironment is associated with poorer prognosis, whereas an immune-activated infiltrate correlates with more favorable outcomes in PDAC.

### 3.3 Identification of ACTG1 as a key upregulated gene closely associated with progression of PDAC

Based on the significant prognostic disparity and distinct immune exhaustion phenotypes observed between the ICI-C1 and ICI-C2 subtypes in our 608-patient training cohort, we aimed to identify differentially expressed genes (DEGs) associated with both PDAC prognosis and immunosuppressive TME. Comparative transcriptomic analysis revealed ACTB, FTL, ACTG1, TMSB10, and TFF1 as the most markedly upregulated genes (**Figure 3A**; **Supplementary Table S5**). In the training cohort of 608 PDAC patients, we stratified patients into two groups according to ACTG1 expression levels. Kaplan-Meier survival analysis demonstrated that patients with low ACTG1 expression had significantly better overall prognosis (**Figure 3B**). Subsequent prognostic assessment of these candidates using TCGA-PAAD data via GEPIA indicated that high expression of ACTG1 was associated with significantly poorer disease-free survival (DFS) compared to low expression (**Figure 3C**), whereas ACTB, FTL, TMSB10, and TFF1 did not exhibit significant associations with patientsurvival (**Supplementary Figures 3A–D**). We further validated the prognostic significance of ACTG1 in an independent PDAC cohort (https://server2.kmplot.com/pancreas). Kaplan-Meier analysis confirmed that high ACTG1 expression was correlated with a marked reduction in DFS (**Figure 3D**).

**Figure 3:**
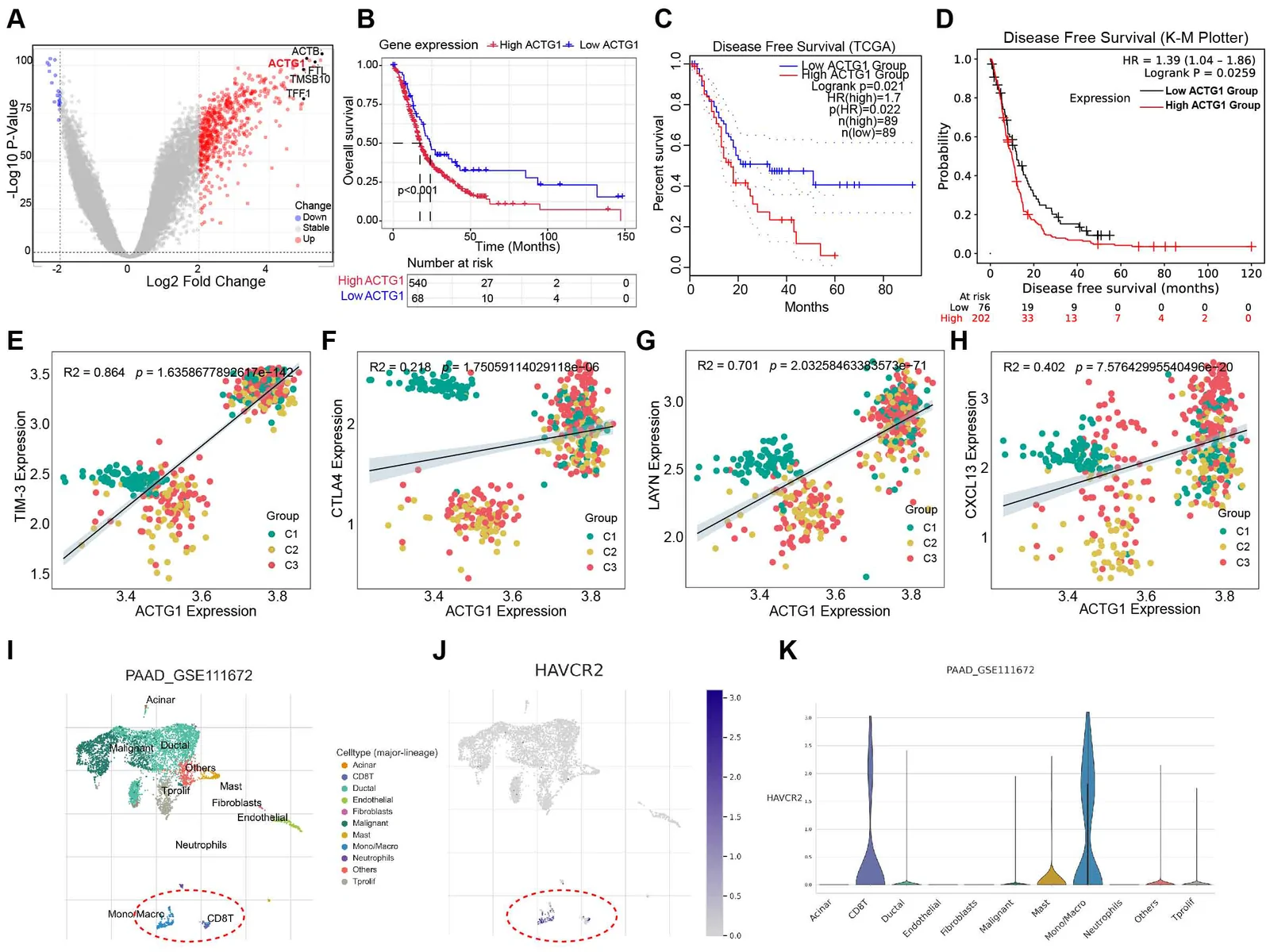
ACTG1 served as a potential prognostic biomarker and correlated with TIM-3 expression on macrophages and CD8^+^ T cells. **(A)** Volcano plot depicting differentially expressed genes (DEGs) following comparative analysis, using a significance threshold of P Value < 0.001 and |Fold Change| > 2. **(B)** Overall survival of the 608-patient PDAC training cohort stratified by ACTG1 expression. **(C)** Disease-free survival (DFS) analysis was performed using the GEPIA2 online platform (http://gepia2.cancer-pku.cn/) in a cohort of 178 pancreatic cancer patients stratified by ACTG1 expression levels. **(D)** DFS analysis was conducted using the Kaplan-Meier Plotter online tool (https://kmplot.com/analysis/) in a cohort of 278 pancreatic cancer patients stratified according to ACTG1 expression level. **(E–H)** Correlation analysis was performed between ACTG1 expression and markers of exhaustion marker (including TIM-3, CTLA4, LAYN and CXCL13) in a cohort of 608 pancreatic cancer patients, using Pearson’s correlation coefficient. **(I–K)** TIM-3 enrichment was a notable feature of the immune landscape in PDAC tumors, as shown by single-cell transcriptomics (GSE111672, TISCH database), particularly within macrophages and CD8^+^ T cells (http://tisch.comp-genomics.org/). Data represent mean ± SEM. To evaluate statistical significance, log-rank test with Bonferroni adjustment was performed to compare survival curves **(B–D)**, Pearson correlation analysis was used to assess the relationship between the two indicated groups **(E–H)**.

### 3.4 ACTG1 promotes PDAC progression by establishing an immunosuppressive microenvironment

Owing to the critical role of immunosuppressive molecules in shaping the TME, we hypothesized that ACTG1 might cooperate with key inhibitory immune regulators. To investigate this potential interplay, we analyzed the correlation between ACTG1 and major inhibitory checkpoints, including PD1, PD-L1, TIM-3, LAG-3, TIGIT, CTLA-4, LAYN, and CXCL13 (18–21) using transcriptomic data from 608 PDAC patients across the GEO and ICGC cohorts. Among the key immune regulators analyzed, ACTG1 expression exhibited significant positive correlations with TIM-3, CTLA-4, LAYN, and CXCL13, particularly with TIM-3 (**Figures 3E–H**; **Supplementary Figures 3E–H**). Based on the strong association between ACTG1 and TIM-3, we further analyzed the expression of TIM-3 on various immune cells within the TME. Analysis of PAAD_GSE111672 single-cell datasets (TISCH)showed that TIM-3 is predominantly expressed on macrophages and CD8^+^ T cells (**Figures 3I–K**). Consistent with this, the cell-type-specific expression pattern was validated across twoindependent TISCH cohorts (PAAD_GSE162708 and PAAD_GSE154778) (**Supplementary Figures 3I–N**).

Based on its significant association with poor prognosis, we further conducted *invivo* experiments using orthotopic pancreatic cancer models. We overexpressed ACTG1 in the49016 (LSL-Kras^G12D/+^, p53^R172H/+^, p48^Cretg/+^) cell line and knocked down ACTG1 expression in the Panc02 cell line (**Supplementary Figure 4A**). Functionally, ACTG1 overexpression significantly promoted orthotopic tumor growth and shortened overall survival. Conversely, ACTG1 knockdown suppressed tumor progression and effectively extended survival (**Figures 4A, B**; **Supplementary Figures 4B–D**).

**Figure 4:**
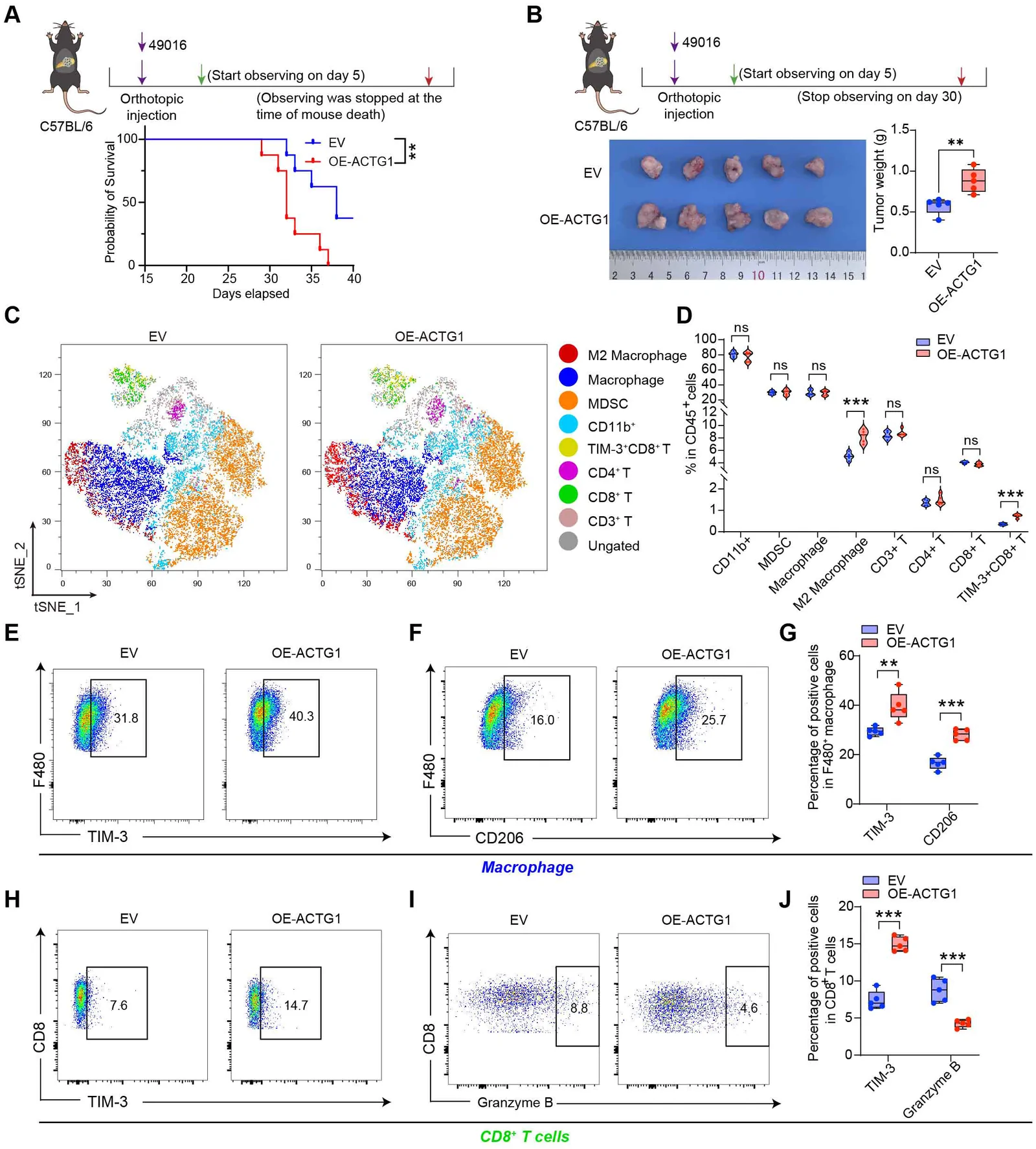
ACTG1 promotes PDAC progression by remodeling the suppressive immune microenvironment. **(A)** Schematic of orthotopic 49016 tumor model in EV and OE-ACTG1 groups in C57BL/6 mice until mice death (up). Kaplan-Meier survival curves showing the survival of mice in EV and OE-ACTG1 (n = 8) (down). **(B)** Schematic of the orthotopic 49016 tumor model (up), with representative images showing tumor growth (left) and the quantitative analysis of corresponding tumor weights for the EV and OE-ACTG1 groups (n = 5). **(C, D)** t-SNE plot of tumor-infiltrating leukocytes overlaid with color-coded clusters and the frequency of clusters of the indicated immune cell subsets, including CD3^+^ T cells, CD4^+^ T cells, CD8^+^ T cells, TIM-3^+^CD8 T cells, CD11b^+^ cells, MDSCs, macrophages and M2 macrophages in the orthotopic pancreatic tumor model across EV and OE-ACTG1 groups, along with the corresponding statistical analysis (n = 5). **(E–G)** Flow cytometric analysis and statistical analysis of percentage of macrophages containing TIM-3^+^macrophages and M2 macrophages from each group (n = 5). **(H–J)** Flow cytometric analysis and statistical analysis of percentage of exhausted CD8^+^ T cells containing TIM-3^+^CD8^+^ T cells and cytotoxic CD8^+^ T cells containing Granzyme B^+^CD8^+^ T cells from each group (n = 5). Data represent mean ± SEM. To evaluate statistical significance, log-rank test with Bonferroni adjustment was performed to compare survival curves **(A)**, a two-tailed Student’s t-test was utilized for **(B)** and One- or two-way ANOVA was utilized to compare two variables **(D, G, J)**. *p < 0.05; **p < 0.01, ***p < 0.001.

Given the positive correlation between ACTG1 expression and an immunosuppressive microenvironment in PDAC, we performed a comprehensive analysis of the orthotopic tumor immune landscape. Interestingly, t-SNE analysis revealed that ACTG1 overexpression did not alter the overall proportions of major immune cell types—including CD3^+^ T cells, CD8^+^ T cells, CD4^+^ T cells, macrophages, and MDSCs (**Figures 4C, D**; **Supplementary Figure 5A**). However, ACTG1 overexpression led to a substantial increase in TIM-3^+^macrophages and M2-like macrophages (**Figures 4C–G**). Conversely, it significantly elevated the proportion of TIM-3^+^CD8^+^ Tcells while reducing the frequency of Granzyme B^+^CD8^+^ T cells (**Figures 4C, D, H–J**).

Together, we demonstrated that ACTG1 drives immune suppression in PDAC not by altering overall immune cell abundance, but by promoting TIM-3-dependent functional exhaustion of CD8^+^ T cells and M2-like polarization of macrophages, thereby facilitating tumor progression.

### 3.5 ACTG1 drives immunosuppression in the PDAC microenvironment via induction of tumor-derived CSF3

To investigate the mechanism by which ACTG1 fosters an immunosuppressive tumor microenvironment, we performed RNA sequencing on orthotopic tumors from EV and OE-ACTG1 groups to identify DEGs. Given the pronounced effect of ACTG1 on TIM-3 expression in TAMs, we integrated macrophage-related genes from Genecards with the DEGs, yielding 25 macrophage-associated DEGs (**Figures 5A, B**; **Supplementary Figure 6A**). Functional enrichment analysis using GO and KEGG revealed significant involvement of these genes in cytokine-mediated signaling pathways, cytokine receptor binding, and cytokine-cytokine receptor interaction (**Figures 5C, D**). Notably, CSF3 stands out among the differentially expressed genes (DEGs), ranking first inthe OE-ACTG1 group compared to the EV group based on both P-value and fold change (**Supplementary Figure 6A**).

**Figure 5:**
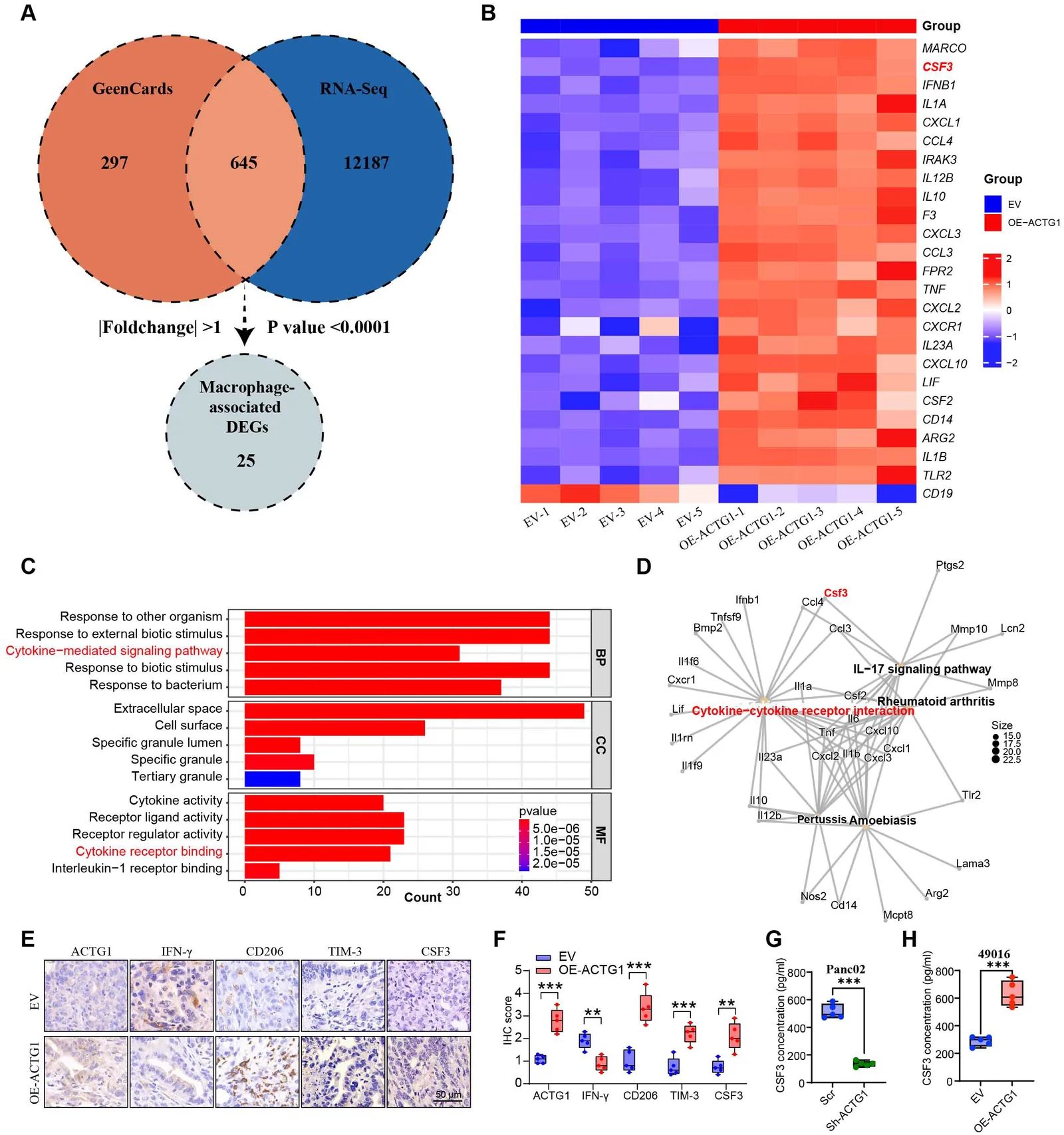
ACTG1 programs an immunosuppressive TME via enhancing CSF3 expression in PDAC. **(A)** Identification of macrophage-associated DEGs. A total of 942 macrophage-associated genes were sourced from Genecards (https://www.genecards.org/). Transcriptomic comparison between the EV and OE-ACTG1 groups of the orthotopic pancreatic cancer model identified 12832 genes. The intersection of these two gene sets was taken, and 25 macrophage-associated DEGs were identified using stringent criteria (P Value < 0.0001 and |Fold Change| > 1). **(B)** Heatmap depicting the expression profiles of the 25 macrophage-associated DEGs in the EV versus OE-ACTG1 groups. **(C, D)** GO **(C)** and KEGG **(D)** analysis of DEGs between the EV and OE-ACTG1 groups. **(E, F)** Representative IHC images **(E)** and corresponding quantitative analysis **(F)** of ACTG1, IFN-γ, CD206, TIM-3, and CSF3 expression in orthotopic pancreatic tumors from EV and OE-ACTG1 groups (n = 5). Scale bars, 50 μm. **(G, H)** The level of CSF3 was analyzed by ELISA in Panc02 and 49016 cells transfected with the indicated plasmids (Scr, Sh-ACTG1, EV, or OE-ACTG1). Data represent mean ± SEM. To evaluate statistical significance, a two-tailed Student’s t-test was utilized for **(F–H)**. **p < 0.01, ***p < 0.001.

Immunohistochemical staining of EV and OE-ACTG1 tumors for immune activation and suppression markers—including IFN-γ, CD206, and TIM-3—showed that ACTG1 overexpression promoted the expression of immunosuppressive markers CD206 and TIM-3, while the expression of immunoactive marker IFN-γ was markedly reduced. Of note, CSF3 was significantly upregulated in OE-ACTG1 pancreatic tumors (**Figures 5E, F**). To determine the effect of ACTG1 on CSF3 secretion, we performed Enzyme-linked immunosorbent assay (ELISA) in pancreatic cancer cell lines (panc02 and 49016). Knockdown of ACTG1 in Panc02 cells significantly reduced CSF3 release, whereas its overexpression in 49016 cells increased it (**Figures 5G, H**). Collectively, these findings identify CSF3 as a key downstream mediator of ACTG1 in shaping the immunosuppressive microenvironment of pancreatic cancer.

### 3.6 ACTG1 upregulates TIM-3 expression on macrophages via the CSF3/CSF3R pathway

To further investigate how tumor-derived ACTG1 upregulates TIM-3 expression in macrophages, we established an *in vitro* co-culture system in which tumor cells were cultured with macrophages. After 48 hours, macrophages were collected for Western blot (WB), immunofluorescence (IF), and flow cytometry analyses (**Figure 6A**). Both WB and IF showed that TIM-3 expression was significantly elevated in J774A.1 macrophages following co-culture with pancreatic cancer cells, and this upregulation was further enhanced when pancreatic cancer cells overexpressed ACTG1 (**Figure 6B**; **Supplementary Figures 7A, B**). A similar trend was observed in Raw264.7 macrophages under the same co-culture conditions(**Supplementary Figures 7C–E**).

**Figure 6:**
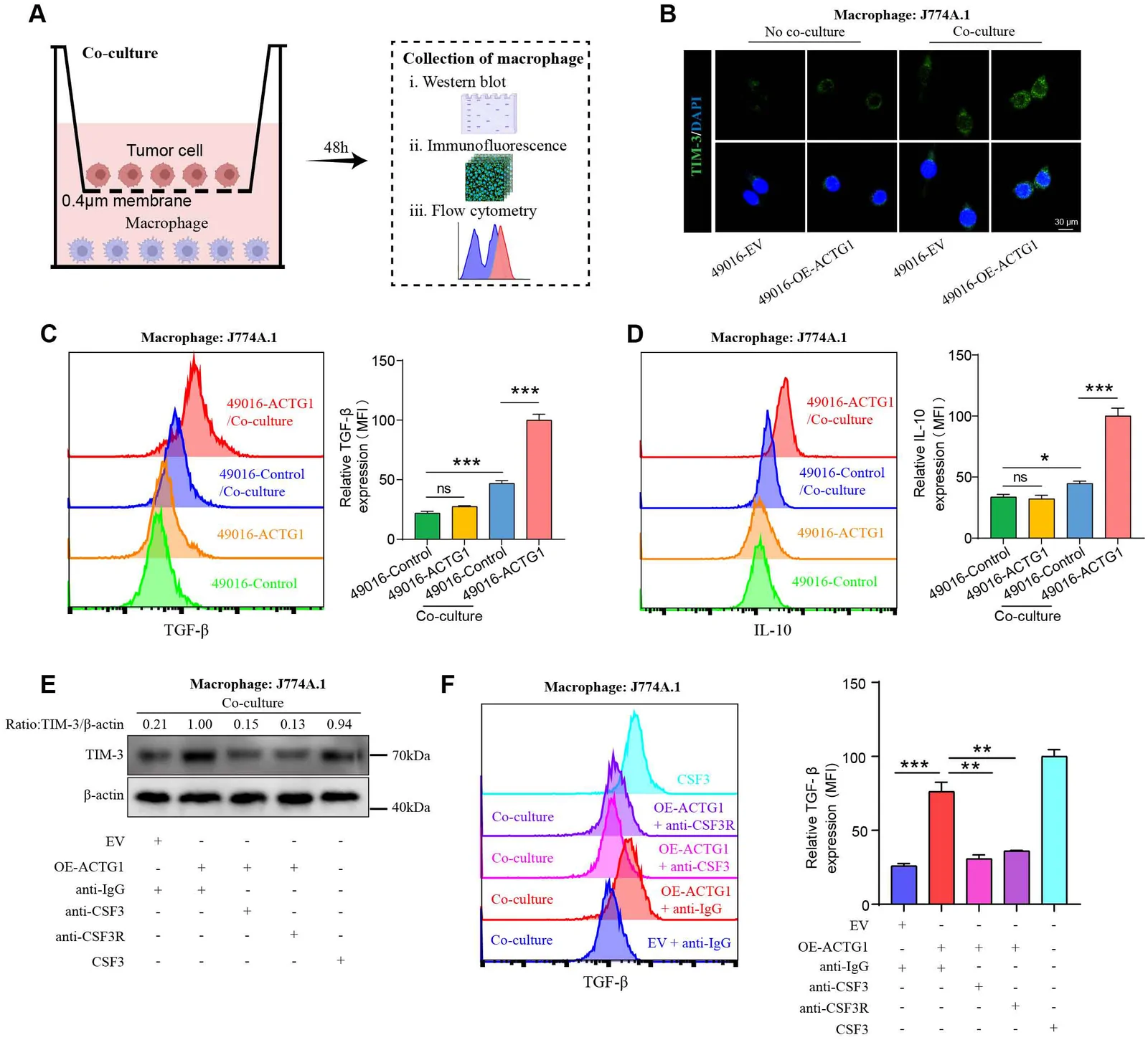
ACTG1 upregulates TIM-3 expression and promotes M2-like macrophages polarization via CSF3/CSF3R axis. **(A)** Schematic diagram depicting the experimental design. The model shows the co-culture of macrophages with tumor cells. After 48 hours, macrophages were harvested and assessed using three complementary methods: western blotting, immunofluorescence, and flow cytometry. **(B)** Representative immunofluorescence images of TIM-3 expression in J774A.1 macrophages following a 48-hour co-culture with or without tumor cells. Scale bars, 30 μm. **(C, D)** Flow cytometry analysis (left) and corresponding quantification (right) of TGF-β **(C)** and IL-10 **(D)** levels in J774A.1 cells after co-culture with 49016-EV or 49016-OE-ACTG1 cells (n = 3). **(E, F)** Western blot analysis **(E)** and flow cytometry analysis **(F)** of J774A.1 cells following co-culture with 49016-EV or 49016-OE-ACTG1 cells treated with specified reagents. For **(F)**, left panels show representative data and right panels show statistical analysis (n = 3). Reagent concentrations: anti-IgG, 1 μg/ml; anti-CSF3, 1 μg/ml; anti-CSF3R, 1 μg/ml; recombinant CSF3, 800 pg/ml. Data represent mean ± SEM. To evaluate statistical significance, One- or two-way ANOVA was utilized to compare two variables **(C, D, F)**. *p < 0.05, **p < 0.01, ***p < 0.001, ns, not significant.

We next assessed M2-like polarization in co-cultured macrophages. Flow cytometry revealed increased expression of the M2-like markers TGF-β and IL-10 in J774A.1 cells co-cultured with pancreatic cancer cells. Overexpression of ACTG1 in pancreatic cancer cells further enhanced TGF-β and IL-10 levels compared to the EV control (**Figures 6C, D**).

Given prior evidence that the CSF3/CSF3R axis plays a key role in shaping an immunosuppressive tumor microenvironment (11, 22), we sought to determine whether this axis directly regulates TIM-3 expression in macrophages. We co-cultured J774A.1 cells with 49016-EV or 49016-ACTG1 cells in the presence of anti-CSF3, anti-CSF3R, or recombinant CSF3. WB analysis confirmed that recombinant CSF3 alone upregulated TIM-3 in macrophages, while blockade of the CSF3/CSF3R axis abolished ACTG1-induced TIM-3 upregulation (**Figure 6E**). Similarly, flow cytometric analysis showed that disruption of CSF3/CSF3R signaling mitigated the promoting effect of ACTG1 on M2-like polarization (**Figure 6F**; **Supplementary Figures 7F, G**).

### 3.7 Genetic suppression of ACTG1 potentiates anti-TIM-3 immunotherapy in PDAC

Given that anti-TIM-3 therapy suppresses pancreatic cancer progression (10), we hypothesized that combining ACTG1 suppression with anti-TIM-3 could enhance antitumor activity. We therefore employed an orthotopic model using Panc02 cells with stable ACTG1 knockdown to provide genetic proof-of-concept for the combination strategy. Stable knockdown of ACTG1 or anti-TIM-3 monotherapy significantly extended the survival of mice with orthotopic pancreatic tumors, and the combination of both resulted in markedly greater survival benefits compared with either treatment alone (**Figure 7A**). Furthermore, the combination of ACTG1 knockdown and anti-TIM-3 antibody significantly suppressed tumor progression relative to monotherapies (**Figure 7B**).

**Figure 7:**
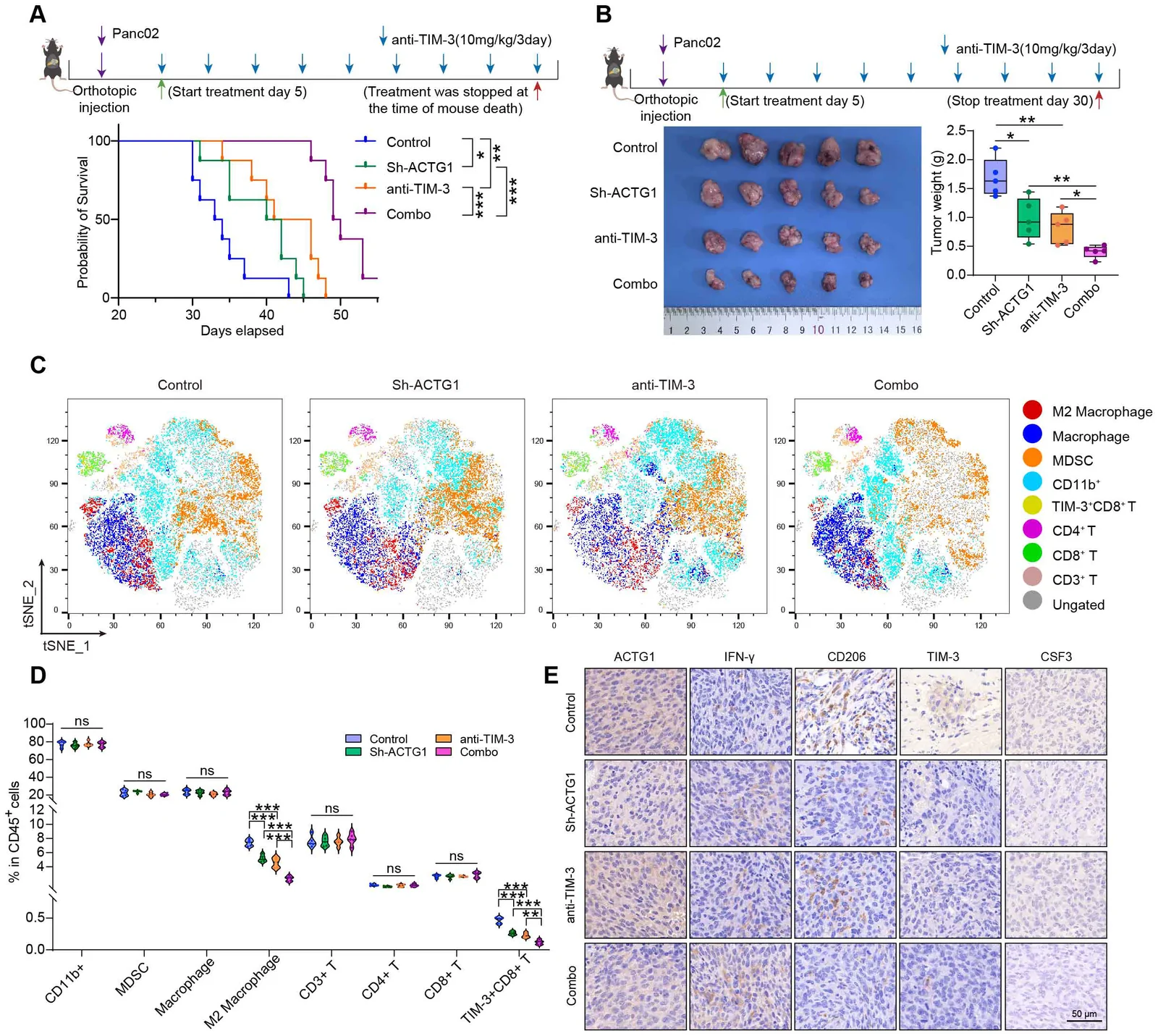
Suppression of ACTG1 markedly enhances the therapeutic efficacy of anti-TIM-3 immunotherapy in PDAC. **(A)** Schematic of orthotopic injection mice models using Panc02-Scr and Panc02-Sh-ACTG1 in C57BL/6 mice treated with IgG or anti-TIM-3 (10mg/kg i.p. injection every 3 days) (n = 8 mice/group) (up); Kaplan-Meier survival curves showing the survival of mice (n = 8) (down). **(B)** Schematic of orthotopic injection mice models using Panc02-Scr and Panc02-Sh-ACTG1 in C57BL/6 mice treated with IgG or anti-TIM-3 (10mg/kg i.p. injection every 3 days) (n = 5 mice/group) (up); Representative images of orthotopic tumor (left) and statistical analysis of tumor weight (right) (n = 5 mice/group). **(C, D)** t-SNE plot of tumor-infiltrating leukocytes overlaid with color-coded clusters and the frequency of clusters of the indicated immune cell subsets, including CD3^+^ T cells, CD4^+^ T cells, CD8^+^ T cells, TIM-3^+^CD8 T cells, CD11b^+^ cells, MDSCs, macrophages and M2 macrophages in the orthotopic pancreatic tumor model across Control, Sh-ACTG1, anti-TIM-3 and Combo groups, along with the corresponding statistical analysis (n = 5). **(E)** Representative IHC images of ACTG1, IFN-γ, CD206, TIM-3, and CSF3 expression in orthotopic pancreatic tumors from Control, Sh-ACTG1, anti-TIM-3 and Combo groups (n = 5). Scale bars, 50 μm. Data represent mean ± SEM. To evaluate statistical significance, log-rank test with Bonferroni adjustment was performed to compare survival curves **(A)**, One- or two-way ANOVA was utilized to compare two variables **(B, D)**. *p < 0.05; **p < 0.01, ***p < 0.001.

Analysis of the immune microenvironment revealed that both ACTG1 knockdown and anti-TIM-3 treatment reduced the proportions of M2-like macrophages and exhausted T cells (**Figures 7C,D**), with the combination producing an enhanced effect beyond that achieved by eitherintervention alone. Flow cytometry analysis of macrophages and CD8^+^ T cell subsetsfurther demonstrated that the combination treatment significantly decreased TIM-3^+^macrophages and increased Granzyme B^+^CD8^+^ T cells compared to single-agent treatments (**Supplementary Figures 8A–D**). Additionally, immunohistochemistry results confirmed that the combination of ACTG1 knockdown and anti-TIM-3 effectively reversed the immunosuppressive pancreatic tumor microenvironment, characterized by a marked increase in IFN-γ and concurrent downregulation of CD206, TIM-3 and CSF3. (**Figure 7E**; **Supplementary Figure 8E**).

In summary, our integrated analysis identifies ACTG1 as a key driver of PDAC progression by reprogramming the tumor immune microenvironment. Elevated ACTG1 in PDAC cells leads to increased CSF3 secretion, which in turn mediates macrophage polarization and TIM-3 upregulation, thereby exacerbating the macrophage-dependent suppression that drives CD8^+^ T cell exhaustion. Importantly, the combination of targeting ACTG1 and anti-TIM-3 therapy inhibits tumor progression and remodels the immune landscape, providing a compelling rationale for combined immunotherapeutic strategies in PDAC (**Figure 8**).

**Figure 8:**
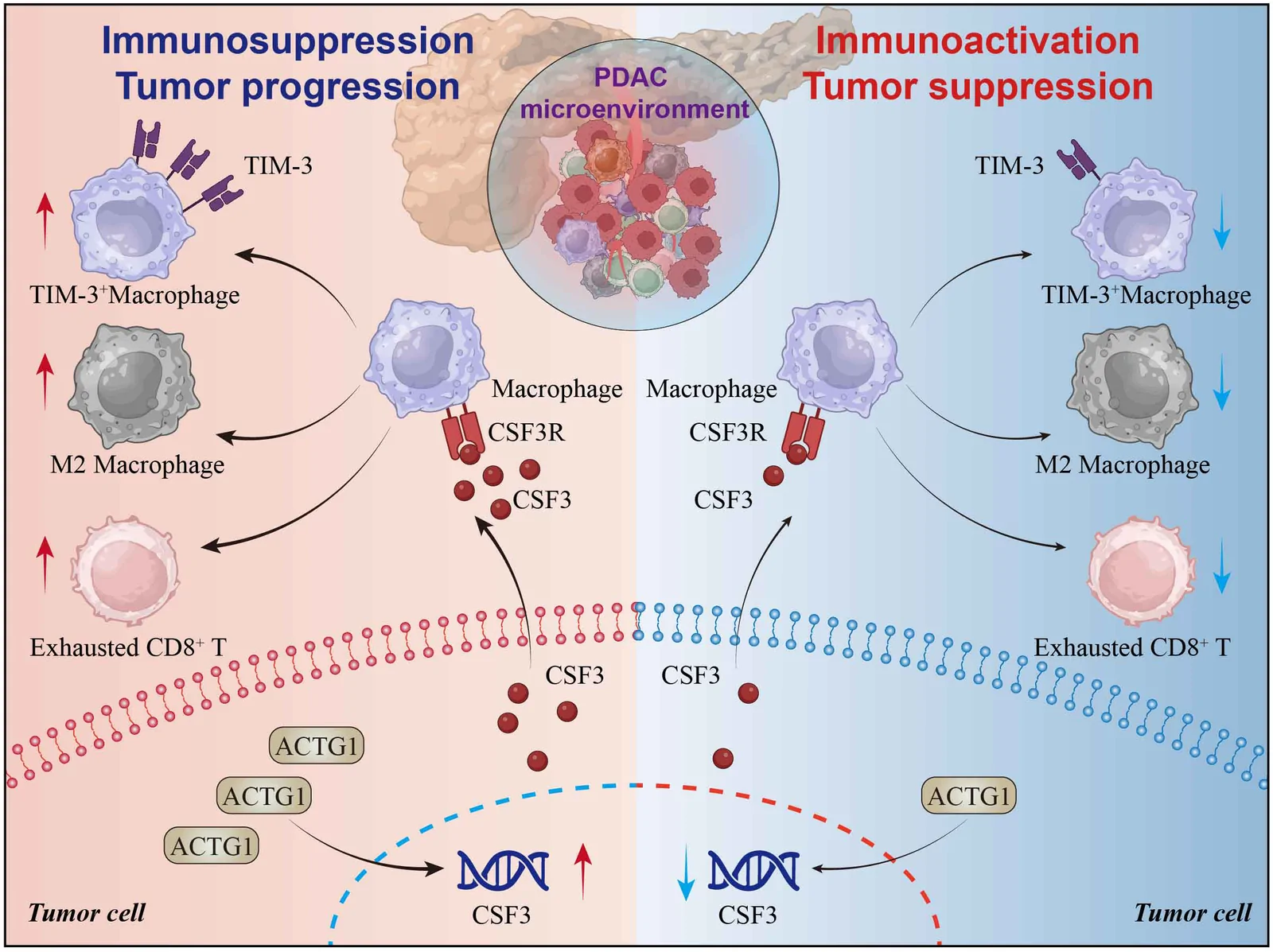
Schematic mechanism depicting how ACTG1 promotes PDAC progression by shaping an immunosuppressive tumor microenvironment. ACTG1 overexpression in PDAC cells promotes CSF3 secretion, which induces macrophages polarization and upregulates TIM-3 expression. This enhances macrophage-mediated suppression of CD8^+^ T cells, leading to their exhaustion. Consequently, the tumor immune microenvironment exhibits increased M2-like/TIM-3^+^ tumor-associated macrophages and accumulated exhausted CD8^+^ T cells, facilitating immune evasion and tumor progression.

## 4 Discussion

PDAC is a highly aggressive malignancy with a dismal prognosis. The immunosuppressive TME of PDAC is considered one of the major drivers of tumor progression. Recent studies in an autochthonous PDAC model demonstrated that combining KRAS* inhibition with IO agents targeting multiple arms of the immunity cycle effectively alleviates the immunosuppressive TME. This combination strategy leads to profound tumor regression and improved long-term survival (23). Although treatment modalities for PDAC include surgery, neoadjuvant chemotherapy, and emerging targeted or immunotherapeutic strategies, their efficacy remains suboptimal, failing to meet urgent clinical needs (3, 24, 25). Therefore, identifying novel therapeutic targets is imperative to inform and advance clinical management in PDAC.

Immune cell dynamics within the TME—their phenotypes and abundance—directly shape clinical outcomes (26). Studies have integrated single-cell immune repertoire sequencing, flow cytometry, and multiplex immunofluorescence to characterize the TME, while applying machine learning algorithms to correlate these features with patient survival. Transcriptomic subtypes have been shown to exhibit distinct clinical outcomes, stemness features, immune landscapes and therapeutic responses, thereby offering a rationale for molecular subtyping in guiding precision oncology (27). Wang et al. reported that HIGD1B is significantly upregulated in gastric cancer tissues and cell lines, and serves as an independent prognostic risk factor. Moreover, patients with high HIGD1B expression displayed increased infiltration of immune cells and were prone to immune evasion and drug resistance following chemotherapy or immunotherapy (28). In PDAC, both intratumoral heterogeneity (ITH) and the TME play pivotal roles in tumor evolution and patient prognosis. The ratio of basal-like ductal cells to immunoreactive cell populations such as cytotoxic T cells significantly influences clinical outcome (29).

ACTG1 is a key gene involved in cytoskeletal composition and regulates multiple biological processes, including cell motility, intracellular transport, and cell communication. Numerous studies have demonstrated that aberrant overexpression of ACTG1 promotes malignant progression in various tumors (30–32). In PDAC, ACTG1 has been shown to facilitate malignant advancement. Cellular and xenograft experiments have confirmed that ACTG1 enhances tumor proliferation *in vitro* and tumor growth *in vivo* (33). Notably, ACTG1 has previously been implicated in bladder cancer pathogenesis, where it exerts a critical regulatory effect on epithelial-mesenchymal transition (EMT) through P4HB splicing and also influences immune cell infiltration (34). Consistent with these findings, our own prior work has shown that ACTG1 enhances EMT in PDAC, creating a positive feedback loop that promotes the polarization of TAMs to an immunosuppressive M2 state through activation of the α2β1/MAPK/ERK signaling cascade (35). Taken together, these observations strongly suggest that ACTG1 contributes to tumor immune exclusion in coordination with the EMT program.

Notably, correlation analysis revealed that among various immunosuppressive molecules examined, TIM-3 exhibited the strongest association with ACTG1. As a well-established immune checkpoint in this malignancy, TIM-3 represents a promising therapeutic target. Supporting this, Chen et al. developed a nanovaccine by coating TIM-3-targeting lipid nanoparticles with antigen-primed dendritic cell membranes (MLP-aTIM-3). This approach not only reversed T cell exhaustion but also fostered tertiary lymphoid structure (TLS) maturation, demonstrating potent anti-tumor efficacy in an orthotopic pancreatic cancer model (36). Consistent with these findings, a phase II clinical trial in advanced pancreatic cancer patients reported that concurrent upregulation of PD-1 and TIM-3 on CD4^+^ and CD8^+^ T cells may constrain T cell responses, suggesting that dual blockade of these checkpoints could restore T cell function (37). Further underscoring the complex role of TIM-3, CD40 agonist monotherapy—despite enhancing CD8^+^ T cell infiltration—triggered a compensatory upregulation of TIM-3, an effect that was reversed when combined with dendritic cell therapy (38). Importantly, TIM-3 exerts distinct direct functions in the two major cell types where it is predominantly expressed: in CD8^+^ T cells, TIM-3 primarily mediates T cell exhaustion and dysfunction, whereas in macrophages it promotes polarization toward an immunosuppressive M2 phenotype, thereby collectively driving the formation of the immunosuppressive tumor microenvironment (38, 39). Therefore, we hypothesize that the ACTG1/CSF3/TIM-3 axis in PDAC primarily mediates the polarization and functional reprogramming of resident or newly recruited macrophages, rather than functioning as a classical chemotactic signaling axis.

In our *in vivo* studies, anti-tumor activity was significantly augmented when anti-TIM-3 was combined with genetic suppression of ACTG1, providing genetic proof-of-concept for an enhanced combined effect of this strategy. This combination therapy is particularly suited for PDAC patients with high ACTG1 expression in tumor cells and elevated TIM-3 expression on tumor-associated macrophages. This patient stratification strategy holds great promise for improving clinical outcomes. However, several challenges remain regarding the use of ACTG1 as a specific therapeutic target. ACTG1 is ubiquitously expressed as a cytoskeletal protein in nearly all cell types, and specific small molecule inhibitors targeting ACTG1 are currently unavailable. Nevertheless, nucleic acid-based therapies represent a promising alternative approach. Small interfering RNAs (siRNAs) can be designed to specifically target unique ACTG1 mRNA sequences, and their antitumor efficacy has been demonstrated in multiple preclinical cancer models (40). Future clinical translation will likely rely on combining si-ACTG1 with advanced targeted delivery systems, including engineered exosomes, lipid nanoparticles, and antibody-oligonucleotide conjugates (AOCs), to achieve tumor-specific delivery of therapeutic agents (41–43).

Cytokines are known to shape the tumor microenvironment, among which CSF3 and its receptor CSF3R play significant roles. Studies have shown that levels of CSF3 and CSF3R are significantly higher in tumor tissues compared to normal tissues. What’s more, CSF3 has been found to suppress T cell production of IFN-γ, and anti-CSF3 treatment was shown to alter tumor T cell infiltration, and IFN-γ response (11). Karagiannidis et al. reported that activation of the CSF3/CSF3R axis can stimulate the production of IL-10 and FoxP3-expressing CD4^+^ and CD8^+^ T cells. In contrast, CSF3R^-/-^ T cells exhibited increased production of IFN-γ and IL-17A, leading to enhanced cytotoxic activity in the TME (44). Furthermore, Ray et al. indicated that elevated CSF3 concentrations can suppress T cell proliferation by upregulating Arg, iNOS, VEGF, IL-6, and IL-1β in CD11b^+^ cells. Knockdown of CSF3 or application of a CSF3-blocking antibody inhibited PDAC tumor growth. *In vivo*, anti-CSF3 therapy combined with gemcitabine was more effective than gemcitabine alone in reducing tumor volume, increasing T cell infiltration, and decreasing Ly6G^+^ cell numbers (11). Furthermore, CSF3 is intrinsically involved in the recruitment of granulocytes and myeloid cells. While our study demonstrates that ACTG1 regulates CSF3-mediated macrophages recruitment, it is important to recognize that CSF3 also recruits other myeloid cell populations, including myeloid-derived suppressor cells (MDSCs) and neutrophils. Guan L et al. showed that CSF3 chemokine mediates radiotherapy-induced recruitment of polymorphonuclear MDSCs (PMN-MDSCs) in tumors expressing Nrf2E79Q via TLR4, thereby enhancing radiotherapy resistance in head and neck squamous cell carcinoma (45). Presicce P et al. previously demonstrated that CSF3 mediates IL-1-dependent upregulation of phosphorylated IRAK1 (p-IRAK1) expression in the amniotic epithelium, which promotes neutrophil infiltration of the chorioamnion-decidua tissue at the maternal-fetal interface (46).

While our study has established that ACTG1 orchestrates the immunosuppressive TME through the CSF3-TIM-3 axis, it is critical to contextualize this mechanism within the unique pathophysiology of PDAC. PDAC is characterized by profoundly poor vascularization and an exceedingly dense desmoplastic stroma, which collectively create a chronically hypoxic tumor microenvironment. Importantly, emerging evidence indicates that multiple key components of the ACTG1/CSF3/TIM-3 signaling axis are functionally linked to metabolic reprogramming, aerobic glycolysis, and adaptive responses to tumor hypoxia (47–49). These signaling pathways engage in intricate crosstalk and synergistically regulate the immunosuppressive microenvironment, thereby driving the initiation and progression of pancreatic cancer.

## 5 Limitations

Our work still has some limitations. First of all, our mechanistic investigations were primarily conducted in orthotopic mouse xenograft models, which cannot fully recapitulate the complex desmoplastic stroma and heterogeneous immune microenvironment of human pancreatic ductal adenocarcinoma; and our *in vitro* co-culture experiments were performed using immortalized macrophage cell-lines (Raw264.7, J774A.1) rather than primary macrophages, which may exhibit different functional phenotypes due to cellular heterogeneity. Secondly, while we validated our findings in multiple public clinical cohorts, the number of patient-derived samples analyzed in our own center was limited, and we lacked paired pre- and post-treatment specimens to assess dynamic changes in the ACTG1/CSF3/TIM-3 axis during therapy. Thirdly, the exact mechanism of ACTG1-mediated CSF3 regulation—whether direct transcriptional control or indirect signaling—remains to be fully defined. Fourthly, our study relied on bulk transcriptomics and immunohistochemistry, and we did not employ spatial transcriptomics or spatial proteomics to directly visualize the spatial colocalization and functional interactions between ACTG1-expressing tumor cells and TIM-3^+^ tumor-associated macrophages *in situ*. Finally, the proposed combination therapy strategy has only been validated in preclinical models, and its clinical safety and efficacy remain to be established in human trials. These limitations highlight important directions for future research to further validate and translate our findings.

## 6 Conclusions and perspectives

In this study, we integrated multi-omics data from ICGC, GEO, and TCGA cohorts, and used ESTIMATE, CIBERSORT, EPIC, and ConsensusClusterPlus to characterize immune subtypes and their prognostic implications in PDAC. Through *in vitro* and *in vivo* experiments, combined with transcriptomic and immunohistochemical analyses, we demonstrated that ACTG1 promotes an immunosuppressive TME via the CSF3/CSF3R/TIM-3 axis, leading to M2 macrophages polarization and CD8^+^ T cell exhaustion. These findings suggest that targeting ACTG1 in combination with TIM-3 blockade may represent a promising therapeutic strategy for PDAC.

Furthermore, providing four key insights for future research. First, it expands our understanding of cytoskeletal proteins beyond structural roles, demonstrating their ability to remotely shape tumor immunity through cytokine secretion. Future work can explore non-canonical immune functions of other actin isoforms and their conservation across cancer types. Second, it establishes a “cytoskeleton modulation + immune checkpoint blockade” therapeutic paradigm. Further studies should optimize si-ACTG1 delivery systems (e.g., engineered exosomes, pancreatic-specific lipid nanoparticles) and evaluate enhanced combined effects with chemotherapy or CSF3R inhibition. Third, it identifies a critical molecular link between tumor cells and macrophages. Spatial transcriptomics/proteomics and organoid co-cultures can dissect the spatial colocalization and paracrine interactions between ACTG1-high tumor cells and TIM-3^+^TAMs. Finally, the proposed “ACTG1+CSF3+TIM-3^+^TAMs” composite biomarker panel provides a framework for patient stratification. Validation in large multicenter cohorts and integration with multi-omics/AI models will enhance predictive accuracy. These findings open new avenues for cross-disciplinary research between cytoskeletal biology and tumor immunology.

## Data Availability

The raw data supporting the conclusions of this article will be made available by the authors, without undue reservation.
