# Supplementary material for: Disruption of ACTG1 potentiates anti-TIM-3 immunotherapy by reprogramming the immunosuppressive microenvironment via the CSF3/CSF3R axis in pancreatic cancer
Source: Front Immunol. 2026 Oct 14;17:1820470. doi: 10.3389/fimmu.2026.1820470 (PMC13713472; doi:10.3389/fimmu.2026.1820470)

Figure. 6E

TIM-3

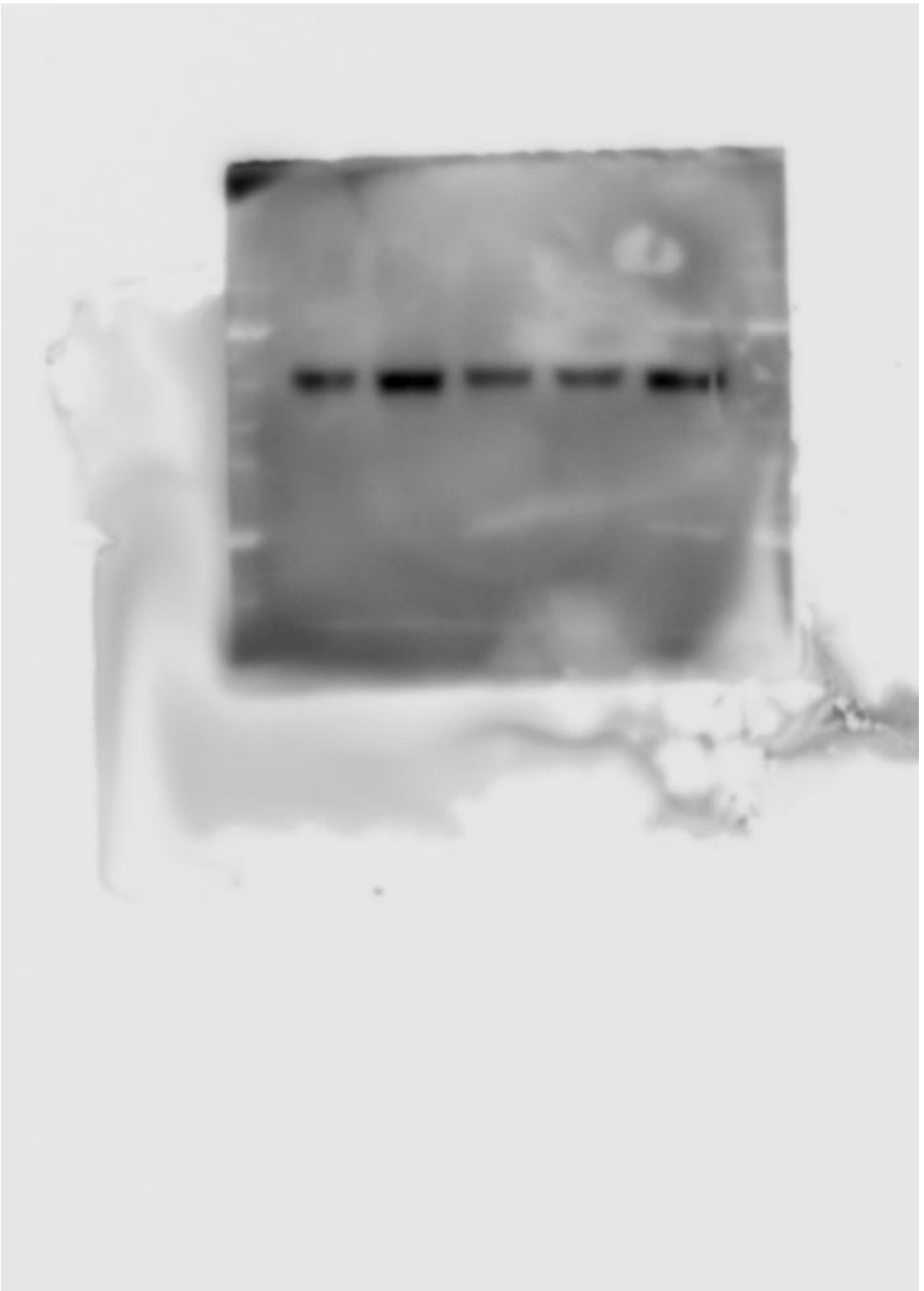

β-actin

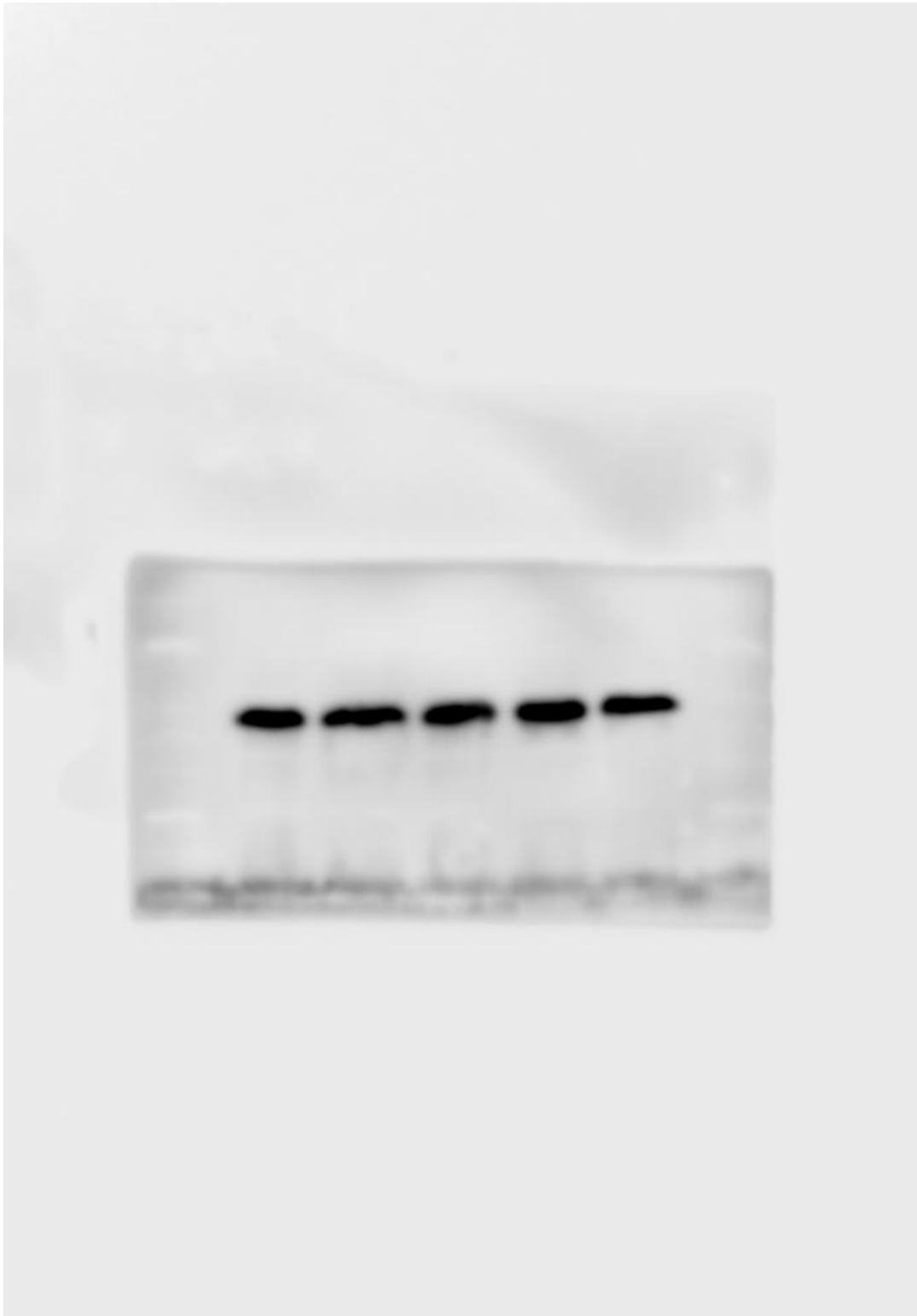

Figure. S3A-shACTG1

ACTG1

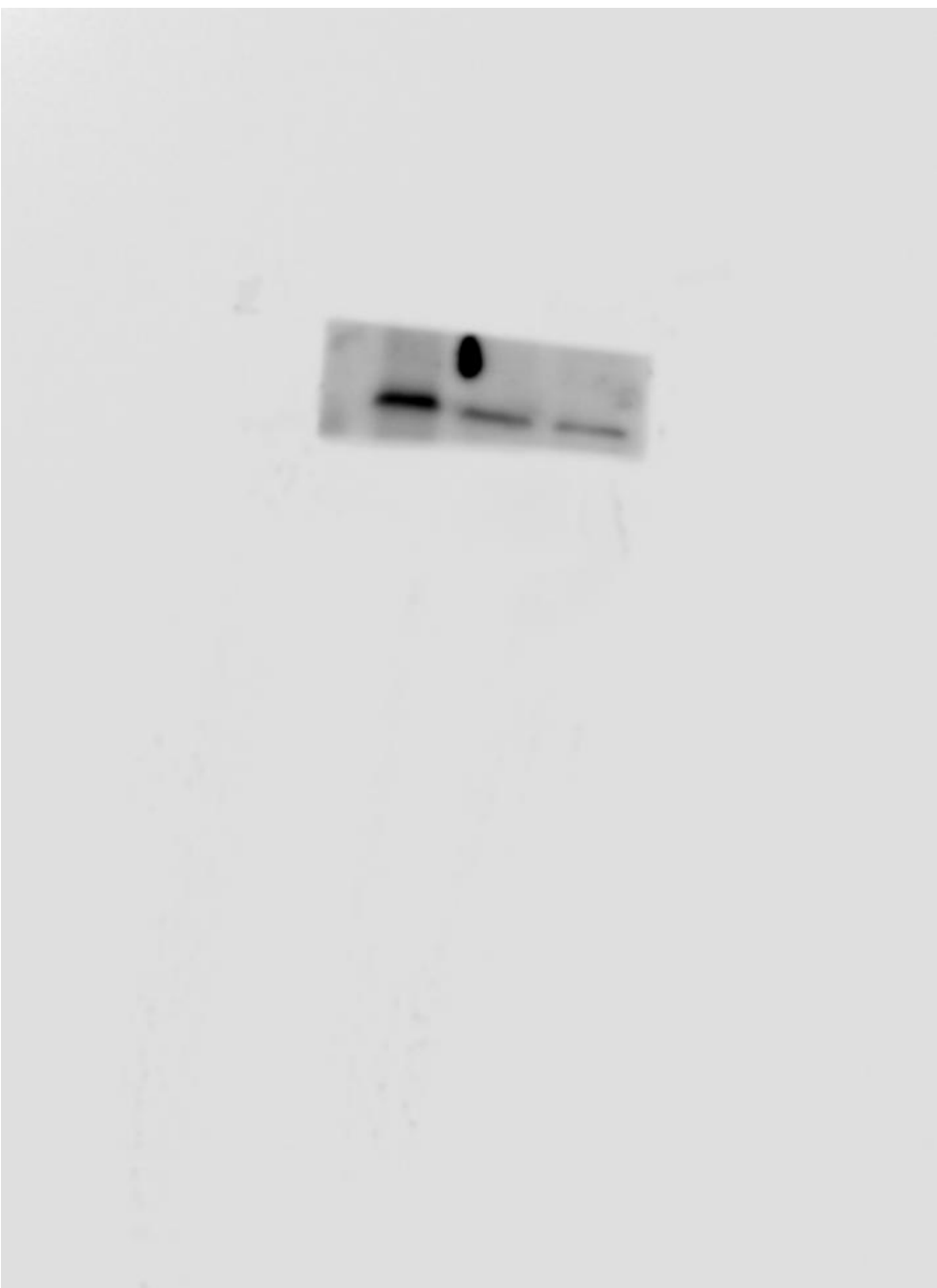

β-actin

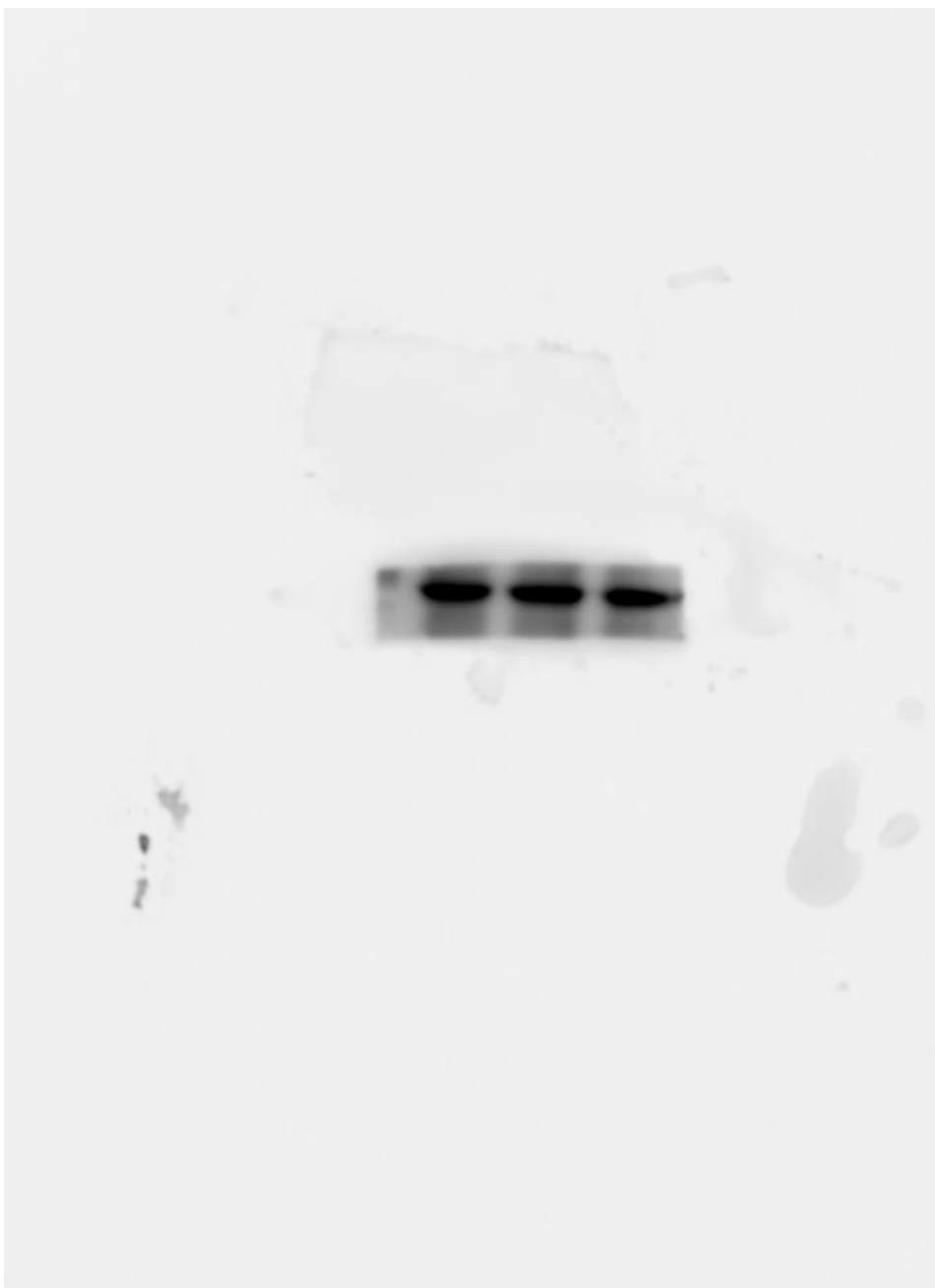

Figure. S3A-OE-ACTG1

ACTG1

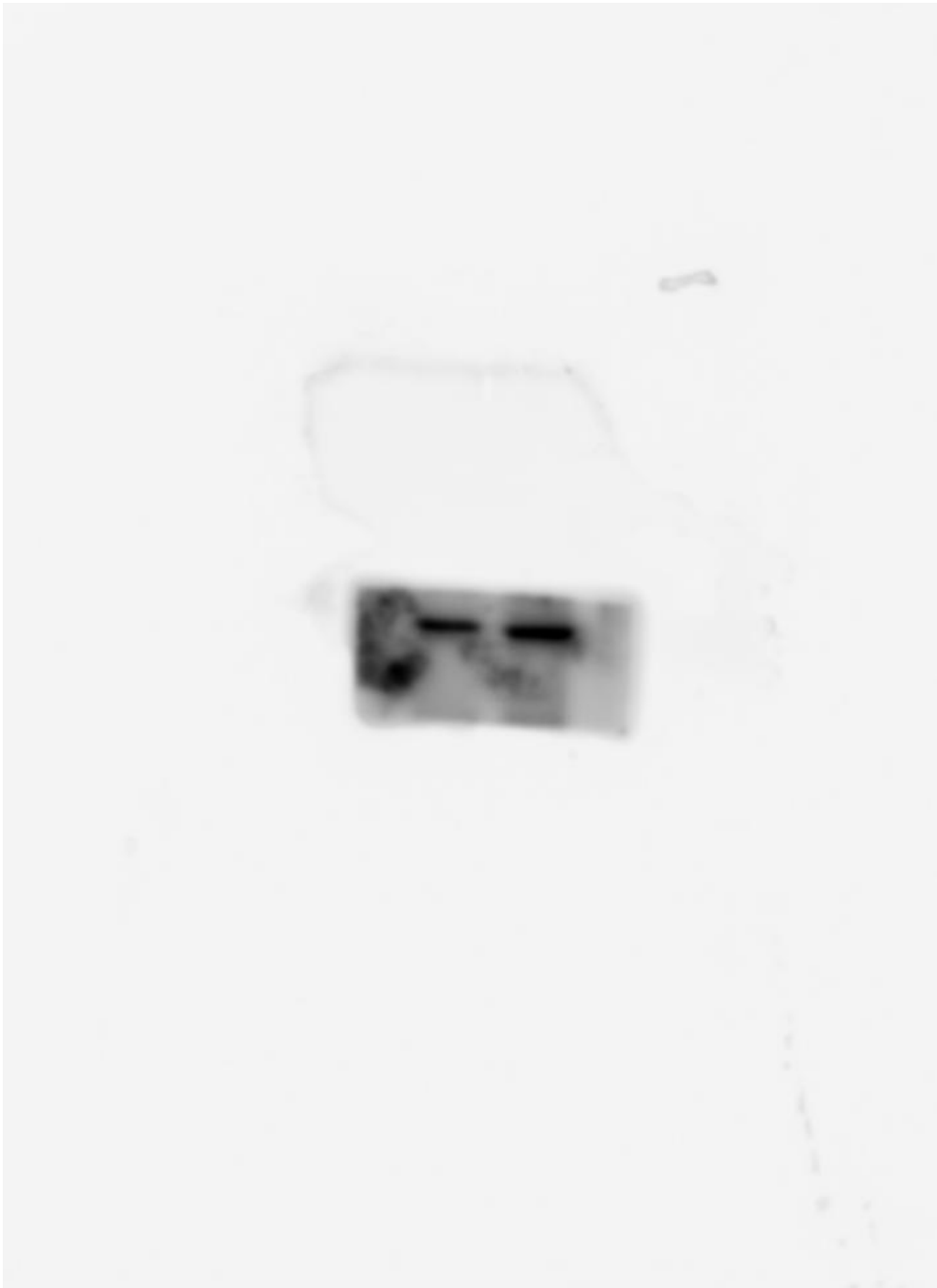

β-actin

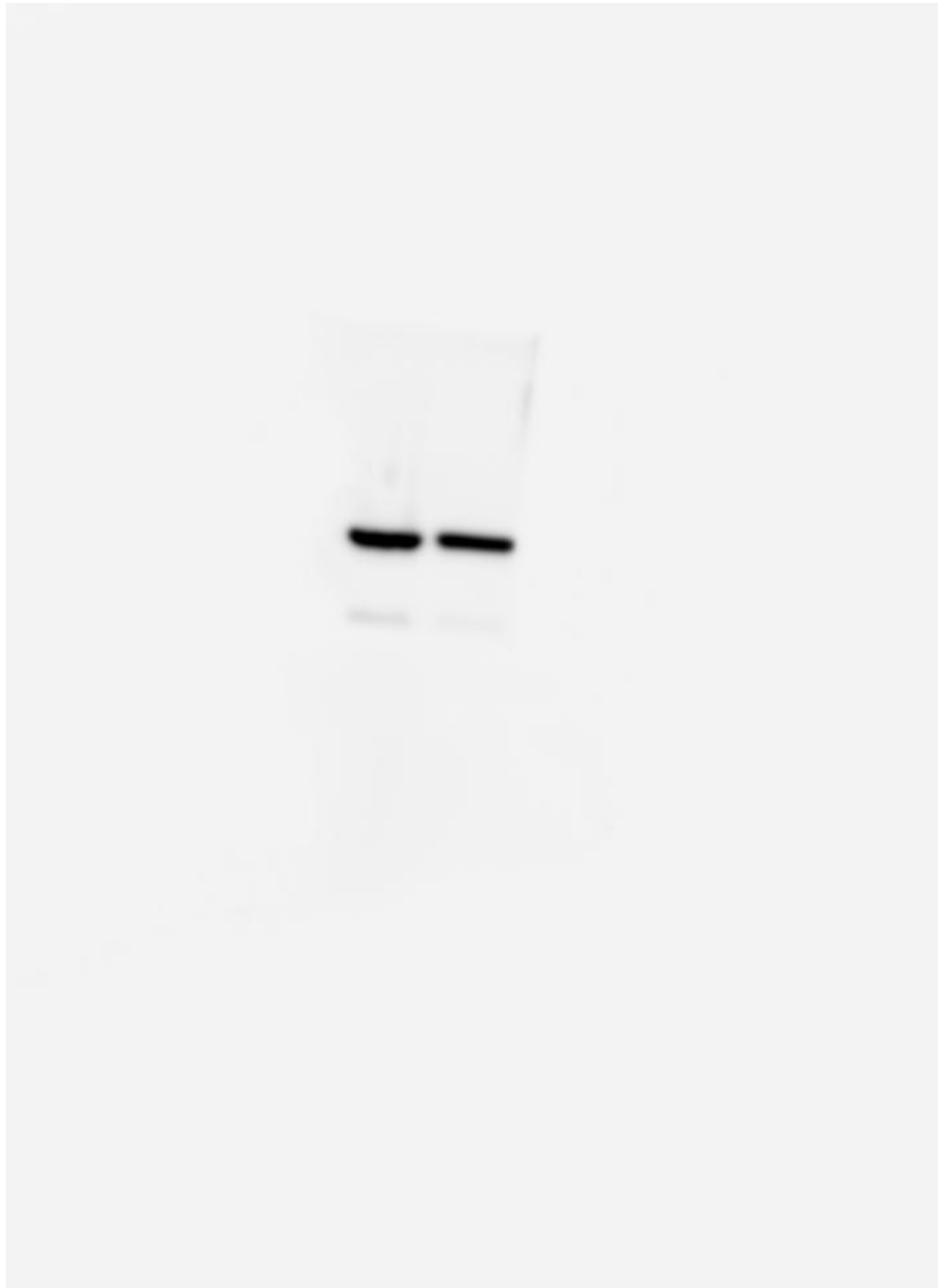

Figure. S5A-J774A.1

TIM-3

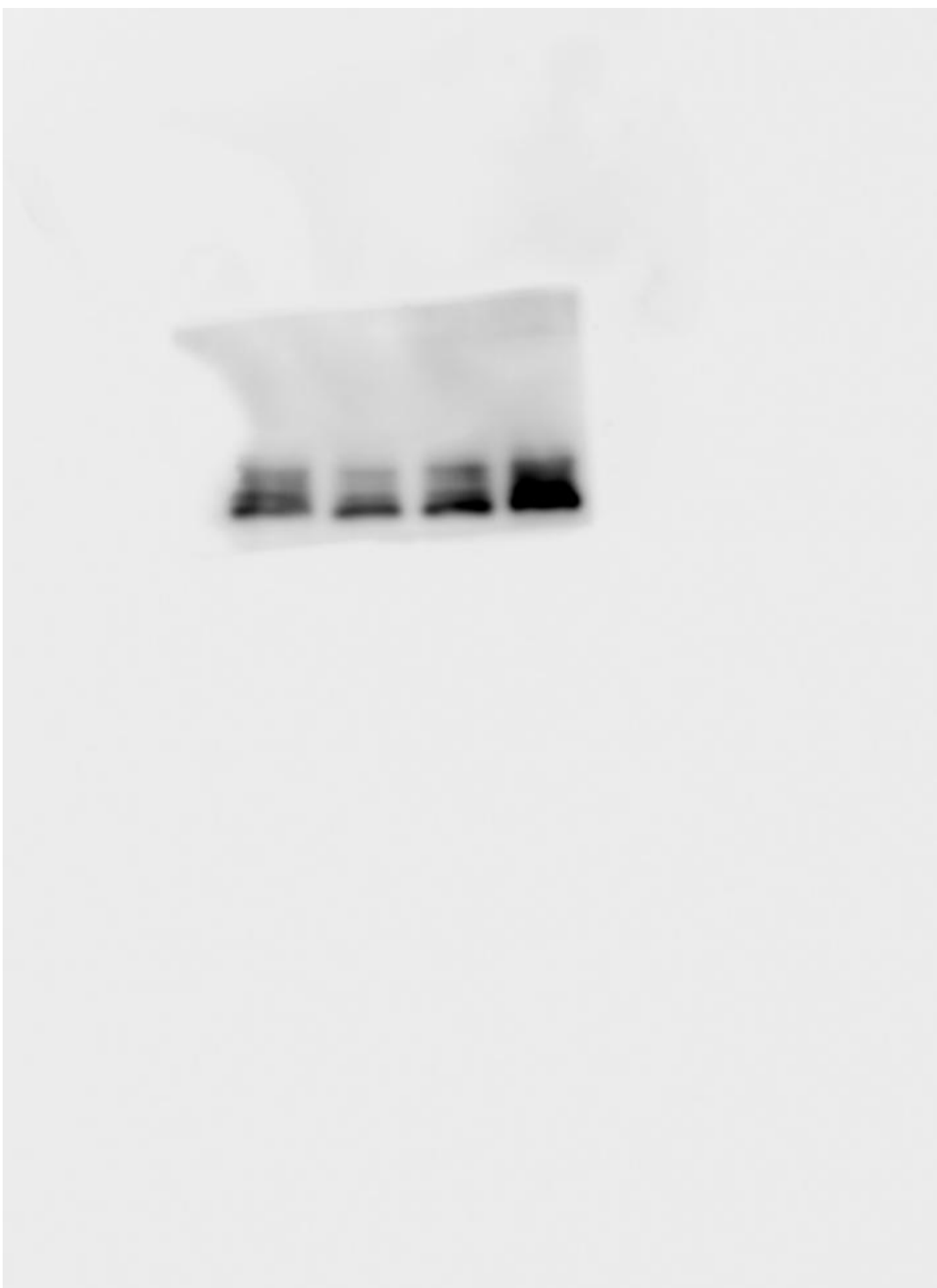

β-actin

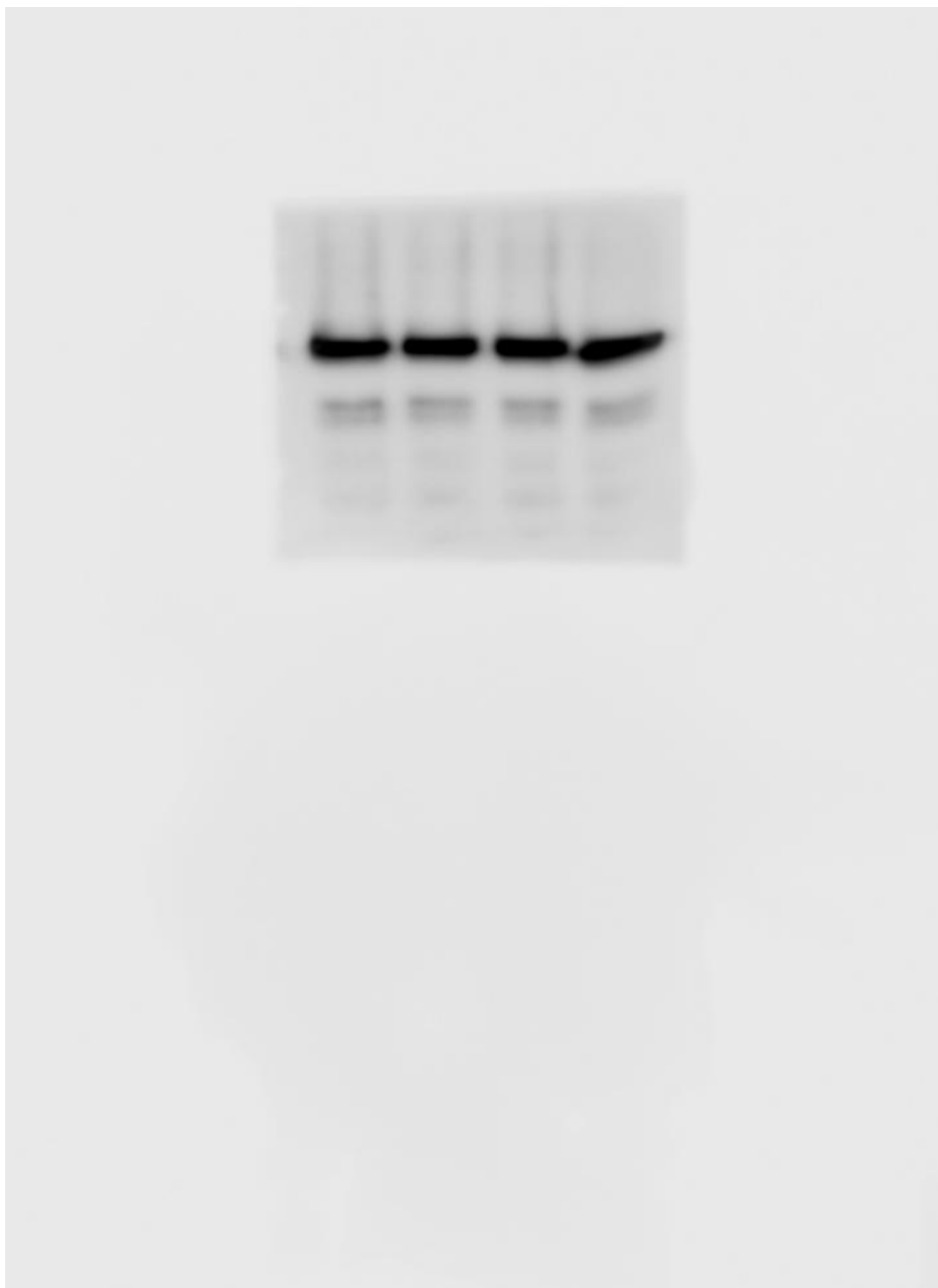

Figure. S5C-Raw264.7

TIM-3

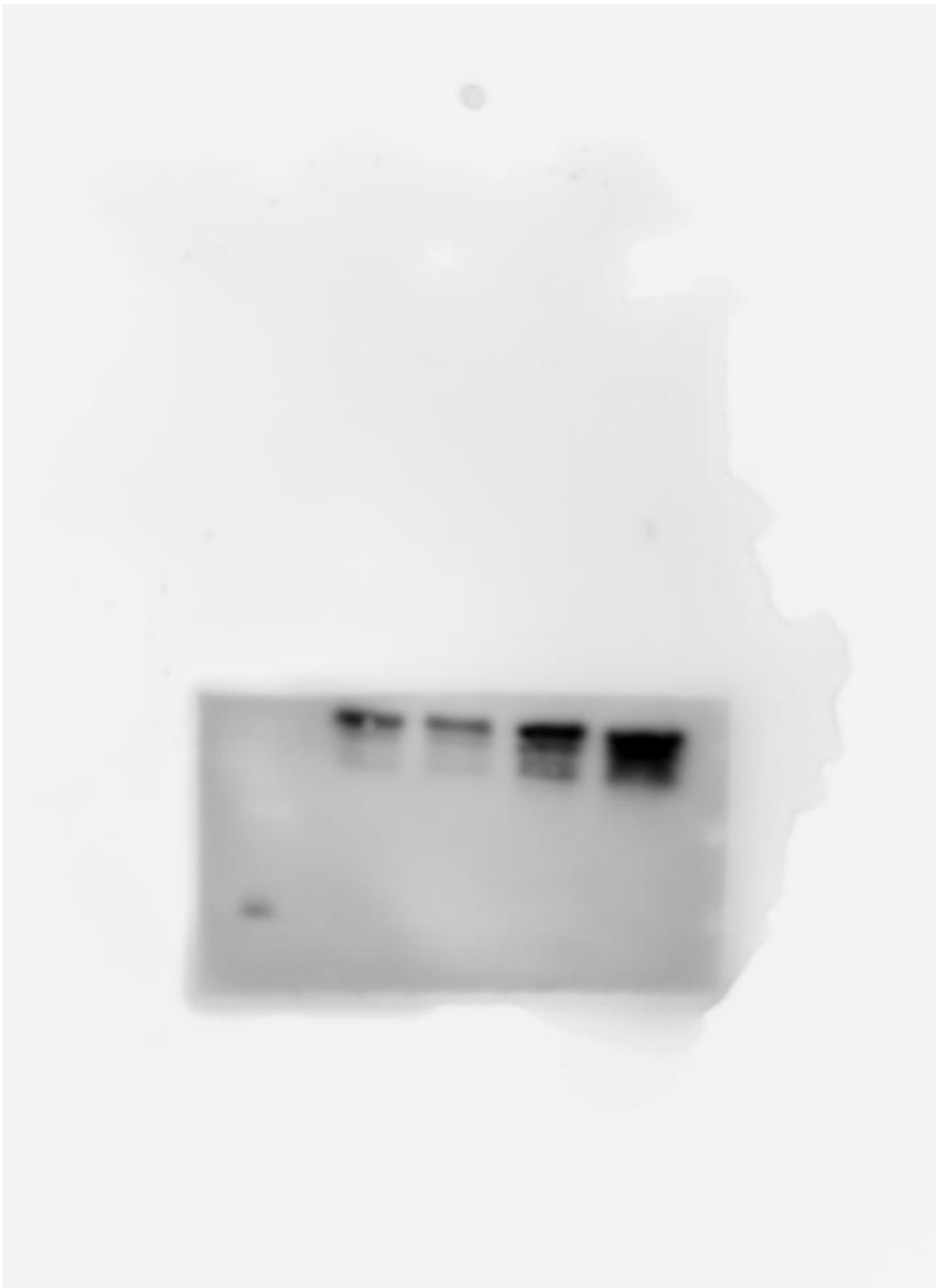

β-actin

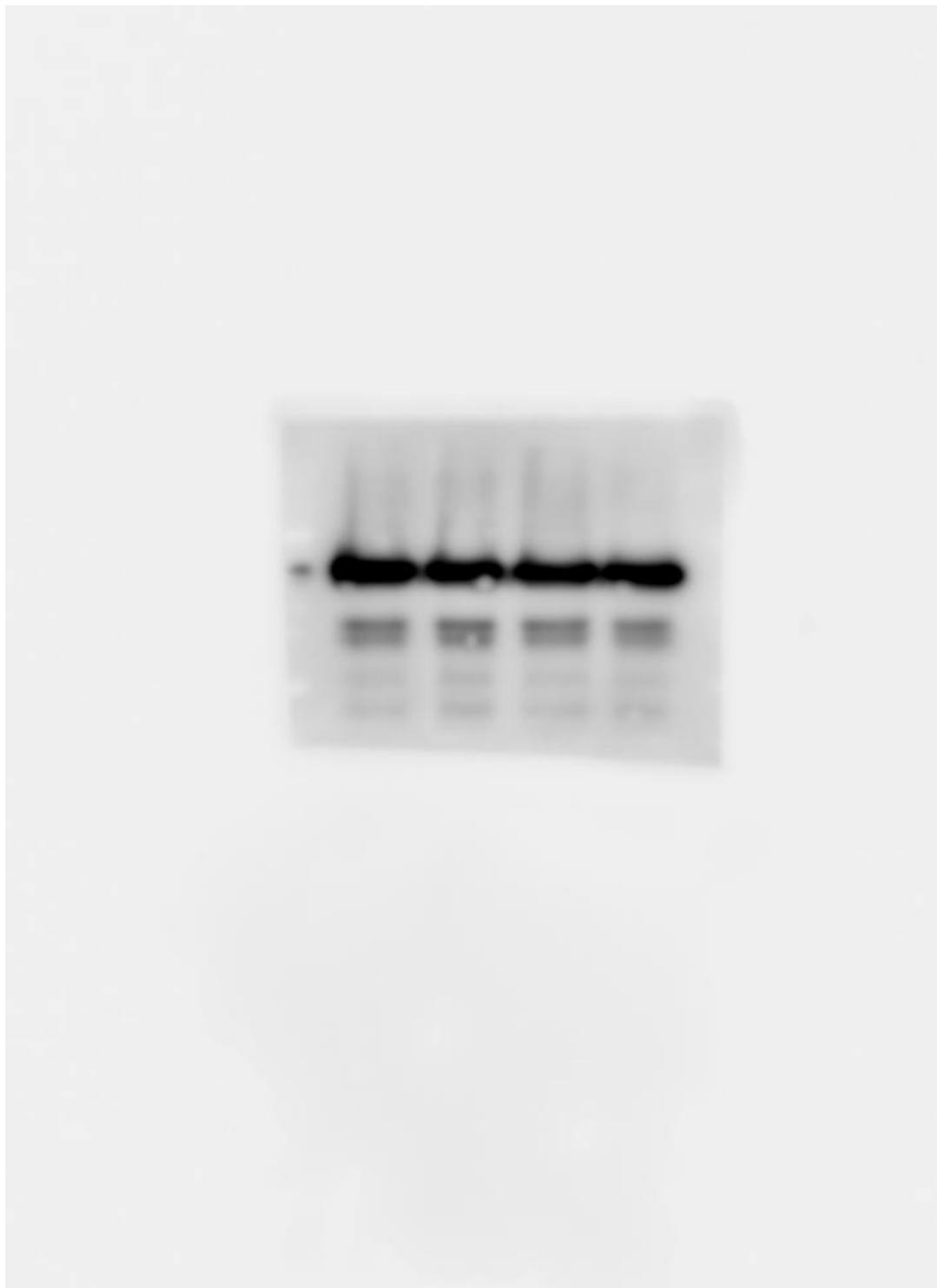

Supplement: Supplementary file 1 [file DataSheet1.pdf]
