# Supplementary material for: Disruption of ACTG1 potentiates anti-TIM-3 immunotherapy by reprogramming the immunosuppressive microenvironment via the CSF3/CSF3R axis in pancreatic cancer
Source: Front Immunol. 2026 Oct 14;17:1820470. doi: 10.3389/fimmu.2026.1820470 (PMC13713472; doi:10.3389/fimmu.2026.1820470)
Supplement: Supplementary file 2 [file Table1.docx]

Supplementary information

Disruption of ACTG1 potentiates anti-TIM-3 immunotherapy by reprogramming the immunosuppressive microenvironment via the CSF3/CSF3R axis in pancreatic cancer

Le Zhu ^1, 2*^, Yu Guo ^1, 2*^, Xinxin Ling ^1*^, Yuqi Tao ^1*^, Shun Wang ^3^, Sanfeng Han ^1^, Meiping Ni ^1^, Huarong Mao ^1^, Jiajun Wu ^1^, Chengjie Zhang ^4^, Ziping Zhang ^1^, Qimeng Chang ^1^, Zhiqiu Hu ^1^, Junmin Chen ^5^, Bingsheng Sun ^6#^, Xiaodi Yang ^1#^, Qiongzhu Dong ^1, 2#^

^1^ Key laboratory of whole-period monitoring and precise intervention of digestive cancer, Shanghai Municipal Health Commission (SMHC), Department of Hepatobiliary and Pancreatic Surgery, Minhang Hospital, Fudan University, Shanghai, China.

^2^ Department of General Surgery, Huashan Hospital & Cancer Metastasis Institute, Fudan University, Shanghai, China.

^3^ Guangzhou Institute of Cancer Research, the Affiliated Cancer Hospital, Guangzhou Medical University, Guangzhou, Guangdong, China.

^4^ College of Acupuncture-moxibustion, Anhui University of Chinese Medicine, Hefei, Anhui, China.

^5^ Traditional Chinese Medicine Department, Minhang Hospital, Fudan University, Shanghai, China.

^6^ Department of Pulmonary Oncology, Tianjin Medical University Cancer Institute & Hospital, National Clinical Research Center for Cancer, Tianjin’s Clinical Research Center for Cancer, Key Laboratory of Cancer Prevention and Therapy, Tianjin, China.

*The authors contributed equally to this work.

**#Correspondence:**

Qiongzhu Dong, PhD,

Key laboratory of whole-period monitoring and precise intervention of digestive cancer, Shanghai Municipal Health Commission, Fudan University, 170 Xinsong Road, Shanghai 200032, China. Tel & Fax: +86-21-54237960; Email: qzhdong@fudan.edu.cn;

Bingsheng Sun, MD,

Department of Pulmonary Oncology, Tianjin Medical University Cancer Institute & Hospital, National Clinical Research Center for Cancer, Tianjin’s Clinical Research Center for Cancer, Key Laboratory of Cancer Prevention and Therapy, Tianjin 300060, China. Email: sunbingsheng@tjmuch.com;

Xiaodi Yang, MS,

Key laboratory of whole-period monitoring and precise intervention of digestive cancer, Shanghai Municipal Health Commission, Fudan University, 170 Xinsong Road, Shanghai 200032, China. Email: yxiaodi1987@126.com;

**Supplementary figures and figure legend**

**Figure S1. ICI subtyping of 608 pancreatic cancer patients via ConsensusClusterPlus and correlation of various immune cells**.

**(A)** Principal component analysis (PCA) showing batch effect correction efficacy in 608 PDAC samples before and after ComBat adjustment following data normalization. **(B)** Consensus matrixes of 608 PDAC training samples for appropriate k value (k = 3), displaying the clustering stability using 1000 iterations of hierarchical clustering. All samples were clustered into 3 clusters. **(C)** The correlation heatmap visualized the comprehensive landscape of immune cell interaction in TME. The correlation coefficient decreased in size from red to blue.

**Figure S2. Validation of ICI subtyping and its correlation with prognosis and immune scores in 177 pancreatic cancer patients**.

**(A)** Consensus matrixes of 177 PDAC samples for appropriate k value (k = 3), displaying the clustering stability using 1000 iterations of hierarchical clustering. All samples were clustered into 3 clusters in the TCGA-PAAD cohort. **(B)** Survival curves were generated using the Kaplan-Meier method, and the log-rank test was applied to assess overall survival differences among the three different ICI clusters. For pairwise comparisons between groups, p-values were adjusted using the Bonferroni method to control the FWER. **(C)** The fraction of tumor-infiltrating immune cells, immune score and stroma score in three ICI clusters.

**Figure S3. TIM-3 expression on macrophage and CD8^+^ T cells in PDAC tumors revealed by single-cell transcriptomics resolution.**

**(A-D)** Disease-free survival (DFS) analysis was conducted using the GEPIA2 online tool (http://gepia2.cancer-pku.cn/) on a cohort of 178 pancreatic cancer patients stratified according to ACTB, FTL, TMSB10 and TFF1 expression levels. **(E-H)** Correlation analysis was performed using the GEPIA2 online tool (http://gepia2.cancer-pku.cn/) between ACTG1 expression and markers of immune exhaustion (including TIM-3, CTLA4, LAYN and CXCL13) in a cohort of 178 pancreatic cancer patients, using Pearson's correlation coefficient. **(I-N)** TIM-3 enrichment was a notable feature of the immune landscape in PDAC tumors, as shown by single-cell transcriptomics (GSE162708, GSE154778, TISCH database), particularly within macrophages and CD8^+^ T cells (http://tisch.comp-genomics.org/).

To evaluate statistical significance, log-rank test with Bonferroni adjustment was performed to compare survival curves **(A-D)**, Pearson correlation analysis was used to assess the relationship between the two groups **(E-H)**.

**Figure S4. ACTG1 knockdown significantly suppresses PDAC progression and prolongs survival in orthotopic Panc02 tumor model.**

**(A)** Western blot of ACTG1, and β-actin in 49016 and Panc02 strain. **(B)** Schematic of orthotopic Panc02 tumor model in Scr and Sh-ACTG1 groups in C57BL/6 mice until mice death. **(C)** Kaplan-Meier survival curves showing the survival of mice in Scr and Sh-ACTG1 (n = 8). **(D)** Schematic of the orthotopic Panc02 tumor model (up), with representative images showing tumor growth (left) and the quantitative analysis (right) of corresponding tumor weights for the Scr and Sh-ACTG1 groups (n = 5).

Data represent mean ± SEM. To evaluate statistical significance, log-rank test with Bonferroni adjustment was performed to compare survival curves **(C)**, a two-tailed Student's t-test was utilized to compare two variables **(D).** *, *p* < 0.05.

**Figure S5. Flow cytometry gating strategies for immune cell subset identification and functional molecule detection.**

**A,** Panel A: Representative gating strategies used to identify all analyzed immune cell populations by flow cytometry, including CD3^+^ T cells, CD8^+^ T cells, Tim3^+^CD8^+^ T cells, CD4^+^ T cells, macrophages, Tim3^+^macrophages and MDSCs; Panels B: Gating strategy for the detection of the cytotoxic effector molecule granzyme B in CD8^+^ T cells.

**Figure S6. Volcano plot of macrophage-related DEGs between EV and OE-ACTG1 groups.**

**(A)** Volcano plot depicting differentially expressed genes following comparative analysis, using a significance threshold of P Value < 0.0001 and |Fold Change| > 1.

**Figure S7. ACTG1 upregulates TIM-3 expression via the downstream CSF3/CSF3R axis, thereby promoting M2-like macrophage polarization.**

**(A)** Macrophages J774A.1 were co-cultured with 49016-EV or 49016-OE-ACTG1 for 48 hours. Western blot analysis of TIM-3 expression in J774A.1 cells. **(B)** Statistical analysis of TIM-3 expression levels in J774A.1 cells with or without tumor cell co-culture. **(C)** Macrophages Raw264.7 were co-cultured with 49016-EV or 49016-OE-ACTG1 for 48 hours. Western blot analysis of TIM-3 expression in Raw264.7 cells. **(D)** Representative immunofluorescence images of TIM-3 expression in Raw264.7 macrophages following a 48-hour co-culture with or without tumor cells. Scale bars, 30 μm. **(E)** Statistical analysis of TIM-3 expression levels in Raw264.7 cells with or without tumor cell co-culture. **(F)** Flow cytometry analysis of J774A.1cells after co-culturing with 49016-EV or 49016-OE-ACTG1 cells treated with indicated reagents (anti-IgG (1 μg/ml), anti-CSF3 (1 μg/ml), anti-CSF3R (1μg/ml), or recombinant CSF3 (800pg/ml)). (**G**) The statistical analysis of the expression level of IL-10 in J774A.1 cells (n = 3).

Data represent mean ± SEM. One- or two-way ANOVA was utilized to compare two variables (**B, E, G**). **, *p* < 0.01, ***, *p* < 0.001.

**Figure S8. Genetic suppression of ACTG1 potentiates anti-TIM-3 immunotherapy by reprogramming the immunosuppressive microenvironment in PDAC.**

**(A-B)** Flow cytometric analysis and statistical analysis of percentage of macrophage containing TIM-3^+^macrphage and M2 macrophage from each group (n = 5). **(C-D)** Flow cytometric analysis and statistical analysis of percentage of cytotoxic CD8^+^ T cells containing Granzyme B^+^CD8^+^ T cells and exhausted CD8^+^ T cells containing TIM-3^+^CD8^+^ T cells from each group (n = 5). **(E)** The statistical analysis of IHC staining intensity for ACTG1, IFN-γ, CD206, TIM-3, and CSF3 in orthotopic pancreatic tumor tissues from Control, Sh-ACTG1, anti-TIM-3 and Combo groups (n = 5).

Data represent mean ± SEM. To evaluate statistical significance, One- or two-way ANOVA was utilized to compare two variables **(A-E)**. *, *p* < 0.05; **, *p* < 0.01, ***, *p* < 0.001.
